# Multimodal machine learning and Raman spectroscopy uncover biochemical pathways of autumnal leaf senescence

**DOI:** 10.1186/s12870-026-08369-1

**Published:** 2026-02-17

**Authors:** Kieran R. Clark, Jarrod L. Thomas, Pola Goldberg Oppenheimer

**Affiliations:** 1 Advanced Nanomaterials Structures and Applications Centre, School of Chemical Engineering, College of Engineering and Physical Sciences, University of Birmingham, Edgbaston, Birmingham, B15 2TT UK; 2 Birmingham Institute of Forest Research, University of Birmingham, Edgbaston, Birmingham, B15 2TT UK; 3 Healthcare Technologies Institute, Institute of Translational Medicine, Mindelsohn Way, B15 2GW Birmingham, UK

**Keywords:** Diagnostics, Ageing, Senescence, Raman spectroscopy, Biomarkers, Mechanisms, Biochemistry

## Abstract

*Quercus robur* – the English oak, undergoes various complex biochemical changes during the process of senescence, one of the most significant being the catabolism of chlorophyll species. The full process of foliaceous ageing remains elusive and largely enigmatic. Here, we employed the use of Raman spectroscopy, density-functional theory calculation, spectrophotometry and colour channel analysis combined with an advanced artificial neural network, to explore senescence-induced biochemical changes to non-venous leaf tissue from *Q. robur* through the process of autumnal senescence. Our analysis demonstrates an increase of Raman-derived crystallinity from 0.185 in non-senescing leaves through to 0.594 in senescing leaves combined with a decrease of the intensity of the 854 cm^− 1^ peak and accompanying increase of the intensity of the 898 cm^− 1^ peak, which in totality suggests that pectin is moving from the α- to β- anomeric form as cellulose moves from an amorphous state into a more crystalline one. Further analysis indicates inconsistencies to the chlorophyll and carotenoid behaviours as leaves move from a non-senescent state to a fully senesced one, which combined with an unexpected increase to the 1215–1320 cm^− 1^ peak region in fully senesced tissue, suggests the presence of new Raman-active bond contributors in fully senesced tissue. Analysis of expected and predicted chlorophyll catabolites indicated the presence of unique bond vibration contributors at 1490 and 1495 cm^− 1^ in all senescence classes and 1510 cm^− 1^ in all senescence classes except in fully senesced tissue, highlighting both the progression of chlorophyll catabolism and potential further breakdown of late-stage catabolites. Classification *via* an advanced artificial neural network showed testing accuracies of 68.18% for non-senescent tissue, 83.40% for visually non-senescing tissue on minimally senesced leaves, 78.15% for visually non-senescing tissue on moderately senesced leaves, 94.15% for minimally senesced tissue, 99.20% for moderately senesced tissue and 100% for fully senesced tissue, yielding an overall differentiation accuracy of 88.50%. Insights obtained herein offer future avenues for both exploratory research and industrial application, namely the exploitation of foliar material for a high-value commercial and medicinal products.

**Supplementary Information:**

The online version contains supplementary material available at 10.1186/s12870-026-08369-1.

## Introduction

The senescence of winter-deciduous tree leaves during autumn results in a striking change of green foliage to a wealth of yellows, oranges, reds and browns as chlorophyll, the pigment provider, degrades whilst the tree reconstitutes nutrients from the leaves into the tree to prepare for winter [1]. This degradation of chlorophyll in leaves from trees like *Quercus robur* (English oak) is marked by complex biochemical changes [2], resulting in the eventual yellowing [3] and browning [4] of the leaves as a plethora of breakdown products are created as the leaf approaches abscission.

**Fig. 1:**
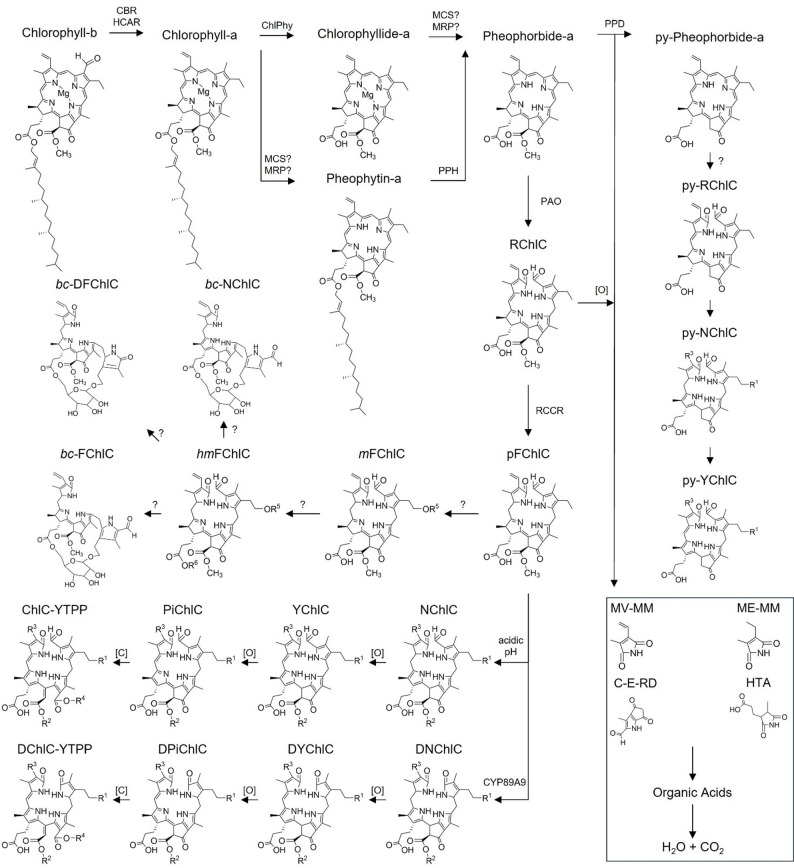
Mechanism of Chlorophyll Catabolism. Enzymatic and non-enzymatic species are outlined. Byproducts are not shown. Side chains (R^1^-R^6^) are summarised in Table 1 MCS: metal-chelating substance; MRP: Mg-releasing protein; PAO: pheophytinase; PPD: pheophorbidase; RCCR: red chlorophyll catabolite reductase; CYP89A9: monooxygenase facilitating conversion of pFChlC to DNChlC; [O]: oxidation [C]: cleavage; MV-MM: methylvinyl maleimide; ME-MM: methylethyl maleimide; C-E-RD: C-E ring derivative; HTA: hematinic acid. Abbreviations starting with D: dioxobilin-type compounds, abbreviations starting with py-: pyro compounds post-decarboxylation of the methyl ester functional group and abbreviations starting with bc-: bicyclic chlorophyll catabolites. For all other abbreviations, see text

Since 1950 chlorophyll-b catabolism has been explored and further understood [5–15] in various plant and algae species and the various catabolites elucidated, however, these are dependent on the species of the plant. For instance, *Hordeum vulgare*, *Brassica napus*, *Cercidiphyllum japonicum* and *Liquidambar styraciflua* produce different chlorophyl catabolites (ChlC) through chlorophyll breakdown [10, 16]. Christ et al. [13] and Kräutler et al. [14] provide detailed overviews of the catabolism of chlorophyll-b during senescence (Fig. 1). Firstly, chlorophyll-b is degraded to chlorophyll-a using the chlorophyll-b reductases (CBRs), non-yellow colouring 1 and non-yellow colouring-like, which both depend on hydroxymethyl chlorophyll-a reductase (HCAR). Following this, chlorophyll-a either undergoes demetallation of the central magnesium of chlorophyll-a through either a Mg-releasing protein (MRP) [17] or a metal-chelating substance (MCS) [18] to produce pheophytin-a; followed by, hydrolysis of the phytyl ester by pheophytinase to produce pheophorbide-a [19]; or, chlorophyll-a undergoes hydrolysis of the phytyl ester by chlorophyllase (ChlPhy) to form chlorophyllide-a and then demetallation of the Mg by a MRP or MCS to form pheophorbide-a. Next, the porphinoid macrocycle is cleaved by pheophorbide-a oxygenase to produce red ChlCs (RChlCs) [20]. Red chlorophyll catabolite reductase (RCCR) then reduces the C20/C1 double bond in RChlCs resulting in primary fluorescent ChlCs (pFChlCs) [21]. From this stage, differentiation of the catabolism process occurs per species, arriving at a complex and varied mixture of fluorescent (FChlCs) and non-fluorescent ChlCs (NChlCs), which are produced when FChlCs are exposed to an acidic pH and non-enzymatic tautomerisation occurs [22]. Further oxidation of NChlCs can form yellow ChlCs (YChlCs) [23] and oxidation of these can produce pink ChlCs (PiChlCs) [24]. There is a secondary catabolism pathway that can occur involving the enzymatic oxidative deformylation of pFChlCs to form dioxobilin-type FChlCs (DFChlCs), first observed in *H. vulgare* [25] and later in *Acer platanoides* [26] and *Arabidopsis thaliana* [27], during which the mechanism was deduced. Similar to FChlCs, these DFChlCs follow non-enzymatic tautomerisation in an acidic environment to form dioxobilin-type NChlCs [28, 29], further oxidation to form dioxobilin-type YChlCs [30] and even further oxidation to form dioxobilin-type PiChlCs [31]. Given the relatively-low detection of PiChlCs and dioxobilin-type PiChlCs in senescent leaves, a further degradation step was recently shown by Li and Kräutler [32] where ring 5 of the PiChlC species is cleaved resulting in yellow bilin-type tetrapyrroles (ChlC-YTPPs).

There are other catabolism pathways which can occur, for example in *Chenopodium album*, enzymatic decarboxylation of the methyl ester functional group in pheophorbide-a by pheophorbidase results in pyropheophorbide-a [33, 34], which is unlikely to decompose further into pyro-FChlCs [14] although pyro- NChlCs and YChlCs are theoretically possible [35]. Additionally, in the leaves of *H. vulgare*, the monopyrroles: methylvinyl maleimide, methylethyl maleimide, C-E ring derivative and hematinic acid were identified alongside simple organic acids, likely through oxidation of the RChlC and pyropheophorbide-a ringed structures [36].

Finally, modified and hypermodified FChlCs (*m*FChlCs and *hm*FChlCs respectively) can be formed from pFChlCs through unknown mechanisms [37, 38], producing structures which are unlikely to oxidise to form further species, such as bicyclo-type ChlCs (bc-ChlCs) found in *Ulmus glabra* [39] and *Vitis vinifera* [40] which have been linked with pathogenic-induced foliar defence. Example *R*-group species in Fig. 1 are given in Table 1, as NCCs oxidise into Y- and PiChlCs, the *R*-group species are retained and thus are not given.

**Table 1:** Example *R*-group species present in known and predicted chlorophyll catabolites where Glsy = glucosyl. For bc-ChlC compounds, the Glsy group connects to both R^5^ and R^6^, denoted as -Glsy-. In NCC and ChlC-YTPP compounds, an ester linkage at R^5^ is denoted as -O-R^5^ in the R^1^ position

*R*^1^	*R*^2^	*R*^3^	*R*^4^	*R*^5^	*R*^6^	ChlC Type	Ref.
OH	CH_3_	CHCH_2_	---	---	---	NChlC	[41, 42]
H	CH_3_	CHCH_2_	---	---	---	NChlC	[22, 42]
H	H	CHCH_2_	---	---	---	NChlC	[43, 44]
OH	CH_3_	CH(OH)-CH_2_OH	---	---	---	NChlC	[45]
-O-R^5^	CH_3_	CHCH_2_	---	Glsy	---	NChlC	[46]
OH	CH_3_	CH(OH)-CH_2_OH	---	---	---	DNChlC	[26]
OH	CH_3_	CHCH_2_	---	---	Glsy	*hm*FChlC	[37]
-O-R^5^	CH_3_	CHCH_2_	---	-Glsy-	-Glsy-	bc-ChlC	[39, 40]
OH	CH_3_	CHCH_2_	CH_3_	---	---	ChlC-YTPP-1	[32]
OH	CH_3_	CHCH_2_	H	---	---	ChlC-YTPP-2	[32]

This breakdown process applies to all senescing tissue, regardless of cause, for instance Mittelberger et al. identified ChlCs in phytoplasma-infected leaves from *Malus domestica* and *Prunus armeniaca* [47] and Moser et al. identified ChlCs in *Ocimum basilicum* leaves following aphid predation or fungal infection from *Botrytis cinerea* [48]. This emphasises the need for a further tool to differentiate between age- or pathogen-induced chlorosis caused by senescence.

It should be noted, however, that other pigment providers such as carotenoids may also degrade through autumnal senescence [5], primarily driven through enzymatic reactions. Further information relating to this specific mechanism is reviewed elsewhere, e.g., [49–52], and more recently by Li, Rodríguez-Concepción and Al-Babili [53]. There may also be changes to the cell wall components of cellulose, hemicelluloses and pectins as the leaf approaches abscission, see Cosgrove [54] and Delmer et al. [55] for detailed reviews as to the structures and functions of these components. Finally, cuticular wax, a thin continuous layer lying above the mesophyll cells could undergo minimal change through autumnal senescence, with Gülz and Müller [56] and Gülz and Boor [57] describing the chemical composition of *Q. robur* leaf wax.

Raman spectroscopy (RS) is a versatile, rapid and non-destructive vibrational spectroscopic method that elucidates a material’s biomolecular structure through the detection of inelastically scattered photons from a sample, producing chemical fingerprints of the sample at the time of measurement [58]. It, thus, becomes possible to examine changes between classes of biological material, for instance, leaf tissue, due to disease [59–61] or plant health [62, 63] but also fruit ripeness assessments [64]; assessments of stress [65] or nutrient content in crops [66]; and pest detection in crops [67]. RS is usually combined with another technique, for instance, Fourier-transform infrared spectroscopy, a different vibrational spectroscopic technique [68, 69], or ultraviolet-visible (UV-Vis) spectrophotometry, an electronic excitation-based spectroscopy [61, 70, 71] to provide complementary data. To assess ChlCs within plant species, liquid chromatography is typically used to elucidate species-specific components with either UV-Vis spectroscopy or mass spectrometry-based detectors followed by nuclear magnetic resonance as a validation tool [23, 43, 44, 72].

Herein, RS is suggested as a viable technique to explore senescence-induced biomolecular changes to non-venous leaf tissue from *Q. robur* through autumnal senescence. These generated leaf spectra were further analysed *via* multiple classification methods including: an advanced artificial neural network (ANN), the self-optimising Kohonen index network (SKiNET), which acts in a supporting role to assigning classifications and peaks-of-interest [73], used according to Clark and Goldberg Oppenheimer [61]. Other classification methods included partial least squares discriminant analysis (PLS-DA), k-means clustering and random forests.

As the chlorophyll catabolite profile has yet to be elucidated for *Q. robur* leaves, using expected and predicted pathways for chlorophyll degradation, simulated Raman spectra were generated using the quantum chemistry program, *ORCA* [74, 75]. RS, used in tandem with *ORCA* computational chemistry simulations of molecules to obtain theoretical spectra, is shown to be a valuable tool for the monitoring and evaluation of senescing leaves, especially in species where chlorophyll catabolites are not known.

Overall, through detailed biochemical analysis of the spectroscopic changes observed as the leaf progresses through different stages of senescence pre-abscission, and comparisons to the *ORCA*-generated simulated spectra of predicted chlorophyll catabolites, we demonstrate Raman spectroscopy’s position as a valuable tool to explore both chlorosis and other biomolecular changes through senescence.

## Methods

### Leaf acquisitions

*Q. robur* leaves, which had emerged in the July growing window, were collected from a site in the September of the same year by cutting the leaf from the branch. *Q. robur* specimens were identified by Doctors Estrella Luna-Diez and Rosa Sanchez-Lucas, both of whom are experts in *Q. robur* research. Although no voucher specimens were preserved, the species is readily identifiable by distinctive foliar morphology, and multiple specimens remain at the collection site. In September this site experienced an average temperature of 16.18 ± 4.16 °C (range, 7.1–23.9 °C) and an average humidity of 75.84 ± 6.92% (range, 57–98%). Chosen leaves were inspected for signs of insect predation or other damage and such samples were discarded to prevent the introduction of an unknown factor influencing the results. The leaf petioles were wrapped in a moistened paper towel, and the leaves were transported in a sealed sample container prior to measurement using Raman spectroscopy, UV-Vis spectrophotometry and photography for red-green-blue (RGB) pixel analysis. The sample collection-measurement delay for Raman spectroscopy was kept consistent at 1 h, to limit the effects of dehydration on the leaf samples. Raman spectra collected within this sample collection-measurement window should show minimal wound-instigated biochemical changes from the excision of the leaf. By 10 min post-excision, jasmonic acid and jasmonic acid-isoleucine are upregulated with a maximum concentration at 30 min post-excision which subsequently declines [76]. Whilst jasmonic acid, a Raman-inactive molecule [77] was not detected, isoleucine is Raman-active [78] so it is not clear where jasmonic acid-isoleucine peaks may lie.

### Raman spectroscopy analysis

Raman spectra were acquired *via* an InVia Qontor Renishaw Raman Spectrometer (Renishaw Plc., UK), equipped with an 830 nm laser with a maximum power output of 500 mW, using a 20x objective with a numerical aperture of 0.4, resulting in a laser spot size of ~ 2.53 μm, and a 1200 lines/mm diffraction grating. The collected leaf samples were secured onto an aluminium foil-wrapped glass slide and areas of the adaxial surface of the leaf blade were measured, taking care to avoid venous tissue, due to the relative lack of chlorophyll in *Q. robur* venous tissue [61]. The leaves were assigned a senescence class based on the visual appearance of the leaf in the ageing process as follows: leaves with no visual de-greening were assigned as non-senescent healthy (HOL), leaves with small patches of yellowing leaf tissue across the blade and brown senesced tissue on the margin as minimally senesced (MinSOL), leaves with large patches of yellowing leaf tissue across the blade and brown senesced tissue on the margin extending within the blade as moderately senesced (ModSOL) and finally leaves fully covered with brown senesced tissue as fully senesced (SOL). Tissue areas with no visual de-greening were also taken from MinSOL and ModSOL tissue resulting in the additional MinSOL-H and ModSOL-H senescence classes. Tissue areas were measured using 10-acquisition 250 × 100 µm^2^ maps with a 50 μm increment per sample for a total of 100 spectra per senescence class. To produce spectra of optimal signal-to-noise ratio, the following parameters were used: a spectral range of 690–1655 cm^− 1^, 5 s acquisition time, ~ 2.5 mW power and 10 accumulations for a total integration time of 50 s/scan. Raman spectra were collected using Wire 5.5 (Renishaw Plc) and cosmic rays were subsequently removed using Wire’s “Width of Features” algorithm and the baseline was subtracted using Wire’s “Intelligent Polynomial” function with a polynomial order of 11 and a noise tolerance of 1.5. Following measurements, the leaf was visually examined for signs of photo- or thermo- degradation, with no visible signs were found for all examined leaves.

### Theoretical Raman spectra generation

Using expected and predicted structures from Fig. 1 and *R*-group species from Table 1, density-functional theory calculations were performed using *ORCA* 5.0.3 [74, 75] to calculate theoretical Raman spectra. Geometry optimisation and frequency calculations were performed using the B3LYP (Becke’s three-parameter hybrid exchange functional combined with the Lee, Yang and Parr correlation functional) hybrid-functional [79–81] in combination with the default-split valence polarisation (def2-SVP) basis set [82, 83] and the “Polar 1” keyword to instigate analytic polarisability calculations. These calculations led to an output file including: the frequency mode, the wavenumber and activity (Å^4^/AMU) [84]. These output files were processed using *ORCA*-enhanced Avogadro [85] to visualise bond vibrations and exporting of the data for further plotting in Microsoft Excel (Office 365 Apps Enterprise, Version 2503).

### U.V spectrophotometry

Leaf tissue of each spectral class was submerged in distilled H_2_O (18.2 MΩ, 10 ml) in a mortar and subjected to pestle grinding for 5 min until paste formation. 2 ml was subsequently transferred to a cuvette. Absorbances (Abs) were determined at 600 nm (A_600_) using a Jenway 6305 UV-visible spectrophotometer following blanking with distilled H_2_O with wavelength accuracy (± 2 nm), and a resolution of 0.1% transmission and 0.001 (a.u.) absorbance.

### Red-Green-Blue pixel analysis

Photographs of leaves from each senescence class were uploaded to ImageJ (Version 1.54) [86] and the whole leaf was selected as a custom region. From each leaf, red, green and blue histograms were produced whereby each histogram represents the colour channel value for each respective colour for each pixel in the custom region per senescence class. A mean colour channel was obtained per image’s histogram and plotted in Microsoft Excel (Office 365 Apps Enterprise, Version 2503).

### Classification methods

The self-organising map (SOM) models were built using the baseline-subtracted non-normalised Raman spectra per class across the same wavenumber range, 690–1655 cm^− 1^, non-normalised spectra were chosen as the spectral intensity changes between classes and their relative importance to the separability of the classes was being observed. These SOM models were built using the SKiNET interface, previously described in Clark and Goldberg Oppenheimer [61], using training data and validated with an unseen testing dataset, whereby this followed the traditional 80/20 split for ANN models, randomly chosen from the baseline-subtracted Raman spectra dataset per senescence class. For each model, the training data was subjected to a 10-fold cross validation and the number of neurons and iterations adjusted until optimal separation was observed. For each SOM, this process was repeated ten times in separate runs to further validate the SOM process. Each SOM provided an output of a confusion matrix whereby the leading diagonal was summed and divided by the sum of all terms to provide an accuracy. SOM discriminant index levels (SOMDIs) were extracted per SOM and plotted on Microsoft Excel (Office 365 Apps Enterprise, Version 2503) for each senescence class.

MetaboAnalyst 5.9 was used to build the PLS-DA model whilst k-means and random forests models were built using custom Python 3.13.3 scripts and these were used to differentiate samples based on class, where appropriate.

### Peak and peak ratio analysis

From the baselined Raman spectra, the ratios, 1003/1525, 1226/1456, 700/1147 and 898/1606 were determined by taking the intensities of the peaks at 700, 898, 1003, 1147, 1226, 1456, 1525 and 1606 cm^− 1^ from each senescence class and applying the formula: ratio a/b = I(a cm^− 1^) / I(b cm^− 1^) where I(x cm^− 1^) refers to the peak intensity at x cm^− 1^. Box and whisker plots of peak ratios and peak distributions through senescence classes were generated using a Python 3.13.3 custom script.

### Statistics

All peak areas, SOM accuracies, intensity ranges, spectrophotometric absorbances, peak ratios and Raman-derived crystallinity values (See Eq. 1) are presented herein as mean ± standard deviation. For all data where *p*-values are stated, statistical significance was set at 5% (*p* = 0.05) unless denoted elsewhere, where *p*-values were determined using Kruskal-Wallis ANOVA followed up with a Dunn’s *post-hoc* test. A Python 3.13.3 custom script was utilised to perform these analyses.

## Results and discussion

Herein, we explore the biochemical changes occurring in senescing leaves. Initially, microscopy and red-green-blue colour channel analyses are performed exploring cellular morphological changes and their respective visual colour changes. Next, the experimental Raman spectra are explored through peak-wise and spectral morphology analyses especially through comparing fully senesced tissue to leaf samples from each stage of senescence and non-senescent subclasses. Following this, simulated Raman spectra of expected and predicted chlorophyll catabolites are outlined, and their assignments are given, with a discussion of expected peak locations in experimental spectra. Next, the data is classified using SKiNET, PLS-DA, k-means and random forests to isolate class-specific differentiators between senescence classes. Finally, spectrophotometry with distribution and ratio analysis of key Raman peaks were explored to find explanations of these class-specific differentiators whilst using the simulated spectra to provide context to results found.

### Microscopy and Red-Green-Blue colour analysis

**Fig. 2:**
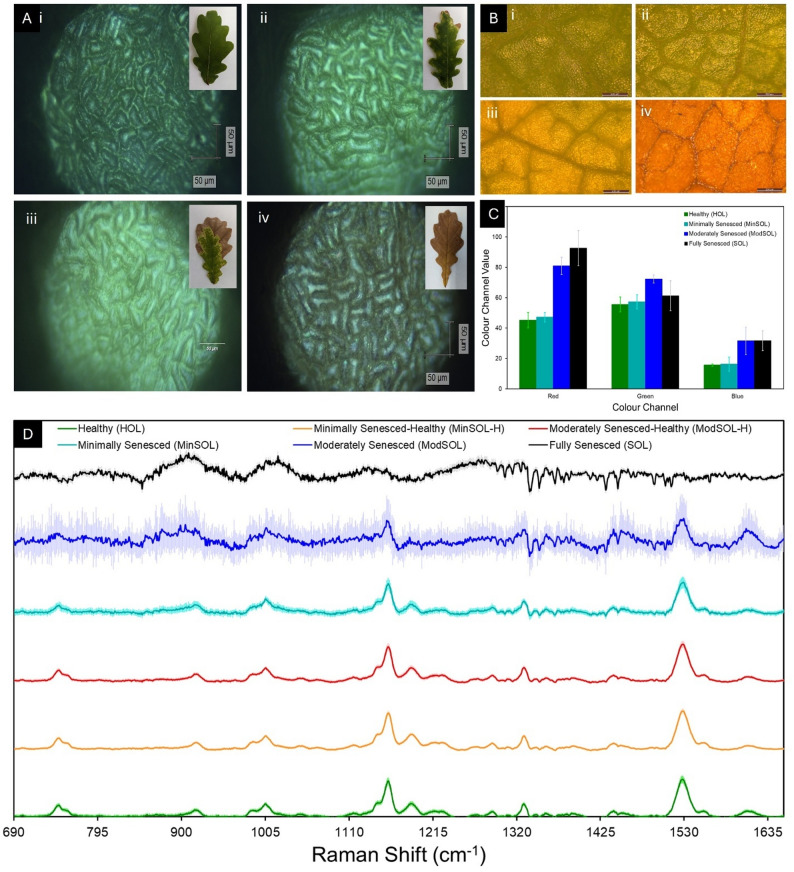
Characterisation and Molecular Profiling of Leaf Classes. **A** Confocal microscopy images of sample classes (i) healthy [HOL], (ii) minimally senesced [MinSOL], (iii) moderately senesced [ModSOL] and (iv) full senescence [SOL], respective digital photography (i-iv, insets). **B** Optical microscopy of corresponding classes: healthy, minimally, moderately and fully senesced (i-iv, respectively), the degree of senescence is highlighted through the notable shift in colour profile. The extent of colour profile variation is demonstrated in **C**. **D** Molecular profiling using Raman spectroscopic fingerprinting of non-senescent, minimally senesced, minimally senesced-healthy [MinSOL-H], moderately senesced, moderately senesced-healthy [ModSOL-H] and fully senesced tissue, normalised by dividing the intensities across the whole wavenumber range by the intensity of the 1003 cm^-1^ peak

For these Raman spectroscopic measurements, mesophyllic tissue separate from the venous tissue was chosen to be measured due to the venous tissue’s resistance to chlorosis [61] (Fig. 2A, i-iv). As seen from HOL (Fig. 2A, i) through to MinSOL (Fig. 2A, ii) to ModSOL (Fig. 2A, iii) and finally to SOL tissue (Fig. 2A, iv), the morphology of these mesophyll cell remains similar despite the leaf becoming gradually less green and browner (Fig. 2A, i-iv insets).

Like confocal microscopy, optical microscopy (Fig. 2B, i-iv) shows similar morphologies for each senescence class, however the optical microscopy does identify the colour change of the tissue. There appears to be little change between HOL (Fig. 2B, i) and MinSOL (Fig. 2B, ii) and this is confirmed in the RGB pixel analysis (Fig. 2C), where there is a non-significant change to the red, green or blue colour channels between these senescence classes (*p* = 1.00 for each colour channel). A non-significant increase to all colour channels from MinSOL to ModSOL (*p* = 0.678, 0.249 and 0.249 respectively) does indicate a yellowing of the tissue, as confirmed in Fig. 2B, iii. Finally, a non-significant increase to the red colour channel (*p* = 1.00), a non-significant decrease to the green colour channel (*p* = 0.537) and no change to the blue colour channel indicates browning of the tissue from ModSOL to SOL tissue, as confirmed in Fig. 2B, iv. Each of these changes is also reflected in the photography of example leaves from each tissue class (Fig. 2A, i-iv insets).

### Spectral fingerprints

Raman spectra collected from different senescence classes included peaks associated with common plant biomolecules, such as carotenoids, cellulose, chlorophyll and lignin; combined with an increasing number of peaks moving from HOL to MinSOL to ModSOL to SOL tissue (Table 2 and Supplementary Table 1), further references of peak assignments for common plant biomolecules can be found in Clark and Goldberg Oppenheimer [87]. Individual, non-normalised spectra for each senescence class can be found in Supplementary Fig. 1.

**Table 2:** Bond vibration profiles and tentative assignments for spectral classes. γ, δ, ν, ρ and ν_s_ refer to, out-of-plane bending, in-plane bending, stretching, rocking and scissoring vibrations respectively

Raman Shift (cm^− 1^)	Bond Vibration	Tentative Assignment	Reference
700	Not Identified	Chlorophyll-a	[88]
744	δ(N–C–C) γ(C–OH)_COOH_	Chlorophyll-a Pectins	[64, 89] [58, 90]
854	C–O–C skeleton	Pectins (α-anomer)	[61, 90]
898	H–C–C, H–CO bending δ(C–C–H), δ(C–OH) δ(CH)_aromatic_	Cellulose (amorphous) Pectins (β-anomer) Xylan	[91, 92] [90] [69]
907	H–C–C, H–CO bending	Cellulose (crystalline)	[92]
917	Not Identified ν(C–O–C)_symm_	Cellulose Lignin	[58, 93]
1003	ρ(CH_3_)_polyene_	Carotenoids	[58, 94]
	ν(C–C)	Phenylalanine	[95]
1020	C–C-coupled ρ(CH_3_)_polyene_	Carotenoids	[96, 97]
1048	ν(C–O), ν(C–C), δ(C–OH)	Cellulose, Lignin	[58, 98]
	ν(CC)(CO)	Pectins	[90]
1128	δ(C–O–C) + δ(C–C)	Xylan	[91]
1147	ν(C_a_N)	Pheophytin-a	[99]
	ν(C_a_N)_II_, ν(C_b_CH_3_), δ(C_a_NC_a_)_I_	Chlorophyll-a	[100]
1157	ν(C–C)	Carotenoids	[58, 94]
	ν(C_a_N)	Chlorophyll-a	[99]
		Pheophytin-a	
	Not Identified	Lignin	
1218	Not Identified	Lignin	[91]
	Not Identified	Xylan	
1226	δ(C_m_H)	Chlorophyll-a	[99]
1272	CH_2_–O–H related	α-D-glucose	[101, 102]
1288	δ(CH_2_, CH_3_)	Aliphatics	[58, 103]
1309	Not Identified	Carotenoids	[104]
1328	δ(CH_3_)	Pectins	[58, 90]
	ν(C_a_N)	Chlorophyll-a	[99]
1343	δ(CH_2_, CH_3_)	α-amyrin	[103]
	ν(C_a_N)	Chlorophyll-a	[99]
1355	δ(CH_2_, CH_3_)	Aliphatics	[58, 103]
	ν(C_a_C_b_)	Chlorophyll-a	[99]
1451	δ(CH_2_, CH_3_)	Aliphatics	[60]
1456	δ(CH_2_, CH_3_)	α-amyrin	[103]
1462	ν_s_(CH_2_)	Cellulose (amorphous)	[92, 105]
1481	ν_s_(CH_2_)	Cellulose (crystalline)	[92, 105]
1494	ν(C_a_C_m_)	Pheophytin-a	[99]
1525	ν(C = C)	Carotenoids	[58, 94]
1606	ν(C–C)_aromatic_	Lignin	[58, 106]
	ν(COO–)_asymm_	Pectins	[90]

Firstly, comparing non-senescent HOL to MinSOL-H and ModSOL-H (Fig. 2D) shows a similarity in the spectral morphology across all wavenumbers. Shared peaks across these classes are primarily from carotenoids (e.g., 1157 and 1525 cm^− 1^), cellulose (e.g., 917 and 1328 cm^− 1^), chlorophylls (e.g., 1188 and 1552 cm^− 1^), hemicelluloses (e.g., 1120 and 1216 cm^− 1^), lignin (e.g., 1188 and 1606 cm^− 1^) and pectin (e.g., 745 cm^− 1^). A significant differentiator between the three non-senescing spectral classes were the intensity counts (Supplementary Fig. 1), with HOL having a maximum intensity of 3375.18 a.u. (± 541.57), MinSOL-H of 5317.59 a.u. (± 542.38) and ModSOL-H of 4239.02 a.u. (± 718.79), with significant differences (*p* < 0.001) between all classes. This intensity difference is likely due to natural variation in the chlorophyll levels, as at 830 nm, chlorophyll-a has residual fluorescence, hence leading to a global change in intensities across all wavenumbers through these senescence classes.

Comparing HOL, MinSOL-H and ModSOL-H to the MinSOL spectra (Fig. 2D) shows similar peak morphology, as noted from the RGB colour channel analysis in Fig. 2B and C. However, new minor peaks, likely from the appearance of early-stage catabolites following the initial degradation of chlorophyll, were present. This degradation becomes apparent in ModSOL tissue (Fig. 2D), where despite some key peaks being present (e.g., the 1003, 1157 and 1525 cm^− 1^ carotenoid peaks and the 1606 cm^− 1^ lignin peak), there are additional peaks present, represented by the standard deviation shading which shows a large variation between samples.

For SOL tissue (Fig. 2D), the spectrum is now dominated by the 907, 1021, 1128 and 1272 cm^− 1^ peaks combined with a multitude of new peaks, akin to the ModSOL spectrum. This denotes major changes to the biochemical milieu within the leaf as it progresses towards the characteristic fully-browned state found in fully senesced leaves pre-abscission. The degradation of chlorophyll-b within the leaf leads to two possible contributors to this changing biochemical profile. From the degradation, early-stage, quasi-mid-stage, mid-stage and late-stage chlorophyll catabolites are being produced; or protein and nucleic acid components within the leaf cells are no longer being masked by the chlorophyll residual signal, or both. Irrespective of cause, new molecules with their own peak-profiles could be apparent in fully senesced non-venous tissue.

Taking the 907 cm^− 1^ peak, associated with crystalline cellulose, and the surrounding 860–970 cm^− 1^ region (Supplementary Fig. 2A), there is no appearance of the peak in either the HOL, MinSOL-H or ModSOL-H, instead this region is dominated by the 917 cm^− 1^ peak, associated with cellulose and lignin-based bond vibrations (Table 2). Moving from MinSOL to ModSOL and finally to SOL spectra, this 917 cm^− 1^ peak intensity remains consistent likely due to the lack of degradation of cellulose moving through senescence, however, the 898 cm^− 1^ peak, associated with amorphous cellulose, pectins and xylan; becomes the dominant contributor in the ModSOL spectrum while the 907 cm^− 1^ peak becomes the notable contributor to the SOL spectrum. This region thus represents a change in the crystallinity of cellulose, moving from an amorphous state to a crystalline one as the leaf approaches full senescence. Integrating using Simpson’s rule shows the relative region area moving from 0.0566 (± 0.0119) to 0.106 (± 0.0506) to 0.168 (± 0.0497) to 0.311 (± 0.0424) a.u. from HOL to MinSOL to ModSOL to SOL (Supplementary Fig. 3A). All *post-hoc* Dunn’s test pairwise comparisons of peak area were significantly different (*p* < 0.001) except for the HOL-ModSOL-H pair (*p* > 0.05), and the MinSOL-ModSOL pair which was significantly different (*p* = 0.00960). This further demonstrates how dominant this region becomes within the senescence class spectra as the leaf approaches full senescence, thus confirming prominent changes whilst simultaneously showing spectral similarity between the HOL and ModSOL-H samples and dissimilarity between the HOL and MinSOL-H classes and the MinSOL-H and ModSOL-H classes.

The 1020 cm^− 1^ peak and the surrounding 980–1055 cm^− 1^ region (Supplementary Fig. 2B) follows a similar pattern. On the HOL, MinSOL-H and ModSOL-H spectra, there is a dominant 1003 cm^− 1^ associated with carotenoid and phenylalanine-based bond vibrations, and a minor 1020 cm^− 1^ peak, associated with carotenoid-based bond vibrations (Table 2). Moving from MinSOL to ModSOL to SOL spectra, this 1020 cm^− 1^ peak gradually becomes more dominant in the region until becoming the dominant peak in this region in the SOL spectrum. As this 1020 cm^− 1^ C-C-coupled CH_3_ polyene rocking peak becomes more dominant compared to the non-C-C-coupled 1003 cm^− 1^ peak, this indicates a degree of further coupling of the CH_3_ polyene rocking mode as the leaf progresses from non-senescent to fully senesced. Integrating using Simpson’s rule shows the relative region area moving from 0.106 (± 0.0112) to 0.146 (± 0.0205) to 0.123 (± 0.0263) to 0.178 (± 0.0233) a.u. from HOL to MinSOL to ModSOL to SOL (Supplementary Fig. 3B), illustrating an increase in region contribution to the overall spectra from HOL to MinSOL, a subsequent decrease and a following increase. For this region, all *post-hoc* Dunn’s test pairwise comparisons were statistically different (*p* < 0.001) except for: the HOL-MinSOL-H, HOL-ModSOL-H and MinSOL-H-ModSOL-H pairs (*p* > 0.05), highlighting further evidence towards the spectral similarity of these classes; and the MinSOL-H-ModSOL and ModSOL-H-ModSOL pairs, showing further evidence towards the spectral dissimilarity of the non-senescent subclasses to non-senescent tissue.

For the 1128 cm^− 1^ peak and the surrounding 1095–1170 cm^− 1^ peak region (Supplementary Fig. 2C), in HOL, MinSOL-H and ModSOL-H, this region is identifiable by a dominant 1157 cm^− 1^ peak associated with carotenoid-based bond vibrations and a 1147 cm^− 1^ shoulder peak associated with chlorophyll-a and pheophytin-a-based bond vibrations (Table 2), alongside a cluster of minor peaks in the 1100–1135 cm^− 1^ range. Moving from MinSOL to ModSOL to SOL tissue, we note a gradual increase in definition of all peaks apart from the 1157 cm^− 1^ peak whose peak definition remains consistent. The 1128 cm^− 1^ peak, associated with xylan-based bond vibrations, contributes more to the region, with 1128 cm^− 1^ becoming the dominant peak in this region in fully senesced tissue, whilst the 1147 and 1157 cm^− 1^ peaks contribute less to the region. These changes potentially indicate an increase of xylan with a loss of carotenoids, chlorophyll-a, pheophytin-a as the leaves progress through senescence. By integrating using Simpson’s rule we see a decrease in relative region area from 0.212 (± 0.0138) to 0.196 (± 0.00872) to 0.132 (± 0.0303) to 0.0945 (± 0.0335) a.u. from HOL through to SOL (Supplementary Fig. 3C), illustrating a lower contribution to the overall spectra suggesting larger changes elsewhere. All *post-hoc* Dunn’s test pairwise comparisons were significantly different (*p* < 0.001), except for: HOL-MinSOL-H, HOL-ModSOL-H and ModSOL-SOL (*p* > 0.05), highlighting spectral similarity between non-senescent subclasses and non-senescent tissue and between moderately senesced and fully senesced tissue; MinSOL-H-ModSOL-H (*p* = 0.0153), further highlighting spectral dissimilarity between the non-senescent subclasses; and finally, ModSOL-H-MinSOL (*p* = 0.00732), highlighting spectral dissimilarity in this region between ModSOL-H and MinSOL tissue and potentially the suggestion of a global indicator of senescence being present.

The most complex change seen, however, is the 1205–1335 cm^− 1^ peak region in which the 1272 cm^− 1^ peak is the most dominant in SOL tissue (Supplementary Fig. 2D). In HOL, MinSOL-H and ModSOL-H tissue, this region is dominated by a 1328 cm^− 1^ peak associated with chlorophyll-a and pectin-based bond vibrations alongside major peaks at 1218, 1226 and 1288 cm^− 1^, associated with lignin and xylan-based bond vibrations, chlorophyll-a-based bond vibrations and CH_2_ and CH_3_ aliphatic-based bond vibrations respectively (Table 2). The MinSOL peak distribution is similar apart from the 1272 cm^− 1^, peak associated with α-D-glucose-based bond vibrations becoming more defined. In ModSOL tissue, the region shifts and a slew of peaks between 1260 and 1295 cm^− 1^ become a dominant feature alongside the 1328 cm^− 1^ peak, with the 1218 and 1226 cm^− 1^ peaks becoming less dominant. Finally, in SOL tissue this 1260–1295 cm^− 1^ peak subregion dominates the spectral region profile, with the 1272 cm^− 1^ peak being the most-dominant peak. The evolution of this spectral region indicates the appearance of many new peak-contributors, especially α-D-glucose, combined with an accompanying decrease in other contributors, such as chlorophyll-a as the leaves progress through autumnal senescence. Integrating using Simpson’s rule, we can see the relative area of this region changing from: 0.0999 (± 0.0120) to 0.104 (± 0.0228) to 0.123 (± 0.334) and finally to 0.341 (± 0.0551) a.u. from HOL to MinSOL to ModSOL to SOL respectively (Supplementary Fig. 3D). This change in relative area clearly further demonstrates the differences between each senescence class in this region, either from molecules degrading or molecules becoming more present in the leaf through autumnal senescence. Through comparing relative areas *post-hoc* Dunn’s test pairwise for this region, all pairs are significantly different (*p* < 0.001) except for: ModSOL-H-MinSOL, as with the prior 1095–1170 cm^− 1^ region, suggests a global indicator in ModSOL-H tissue which is present in MinSOL. HOL-MinSOL, MinSOL-H-ModSOL-H, MinSOL-H-ModSOL and ModSOL-H-ModSOL are not significantly different (*p* > 0.05), suggesting spectral similarity between the non-senescent subclasses, between these subclasses and moderately senesced tissue and between non-senescent and minimally senesced tissue, highlighting further evidence of a global indicator of senescence being present in the leaf.

### Simulation of chlorophyll catabolite Raman spectra

Simulated Raman spectra for each chlorophyll catabolite were collated and truncated to the range of interest, 690–1655 cm^− 1^, are found in Supplementary Fig. 4. Following truncation, the five most intense peaks were isolated per catabolite, ordered from highest activity to lowest. Any catabolite peak which had an activity of < 30 a.u. was discounted as they were determined to result in peaks which would be indistinguishable from noise in the experimental spectra. From these remaining peaks, vibrations with the highest force vectors were determined to be the bond vibration(s) responsible for the peak found. The filtered peaks, their respective catabolites and bond vibrations are found in Table 3 showcasing a range of catabolites from early-, mid- and late-stage in the chlorophyll catabolism process. Most of the resultant catabolite peaks (29/45) were from early-stage, 7/45 were from mid-stage and 9/45 were from late-stage catabolites, predominantly from chlorophyll-b, chlorophyllide-a, pheophorbide-a, pheophytin-a and py-pheophorbide-a.

**Table 3:** Filtered bond vibration profiles for simulated catabolites. ω, ρ, ν_s_, ν and γ refer to wagging, rocking, scissoring, stretching and out-of-plane bending vibrations respectively. Superscripted 1, 2 or 3 indicate whether the catabolite is early-, mid- or late-stage in the catabolism process. Letters in bold **(A**, **B**, **C**, **D** or **E)** refer to rings A-E respectively and X-Y indicates the C-H bond between rings X and Y. Italicised groups specify group-specific vibration

Raman Shift (cm^− 1^)	Simulated Catabolite	Bond Vibration
881	Pheophorbide-a^**1**^	ω(CH)_**B−C**_, ω(CH)_**A−B**_
1107	py-Pheophorbide-a^**1**^	ω(CH)_**D**_
1138	Pheophytin-a^**1**^	ω(CH)_**A−D**_, ω(CH)_**D**_
1160	py-Pheophorbide-a^**1**^	ω(CH)_**A−D**_
1321	Pheophytin-a^**1**^	ρ(CH_2_)_Phytol_
1346	Pheophytin-a^**1**^	ω(NH)_**A**_, ω(CH)_**D**_
1366	Chlorophyllide-a^**1**^	ρ(CH_2_)[(**D**)-*CH*_*2*_-*CH*_*2*_-COOH], ω(CH)_**D**_
1369	Chlorophyll-a^**1**^	ρ(CH_2_)[(**D**)-*CH*_*2*_-*CH*_*2*_-COOPhytol], ω(CH)_**D**_
1374	Pheophorbide-a^**1**^	ρ(CH_2_)[(**B**)-*CH*_*2*_-CH_3_], ρ(CH_3_)[(**B**)-CH_2_-*CH*_*3*_], ρ(CH_3_)[(**B**)-*CH*_*3*_], ρ(CH_3_)[(**C**)-*CH*_*3*_]
1378	py-Pheophorbide-a^**1**^	ν_s_(CH_3_)[(**C**)-*CH*_*3*_], ω(NH)_**C**_, ω(CH)_**D**_
1396	Chlorophyll-b^**1**^	ρ(CH_2_)[(**D**)-*CH*_*2*_-CH_2_-COOPhytol], ω(CH)_**D**_, ρ(CH_3_)[(**A**)-*CH*_*3*_]
1406	Pheophorbide-a^**1**^	ρ(CH_3_)[(**C**)-*CH*_*3*_], ρ(CH_3_)[(**D**)-*CH*_*3*_], ρ(CH_2_)[(**A**)-CH=*CH*_*2*_]
1430	Chlorophyll-b^**1**^	ν(CH_3_)[(**A**)-*CH*_*3*_]
1435	Chlorophyllide-a^**1**^	ν(CH_3_)[(**A**)-*CH*_*3*_], ν(CH_3_)[(**D**)-*CH*_*3*_]
1445	ChlC-YTPP-1^**3**^	ρ(CH_2_)[(**D**)-*CH*_*2*_-CH_2_-COOH], ν(CH_3_)[(**D**)-*CH*_*3*_], ω(CH)_**A−D**_, ω(NH)_**A**_
1460	NChlC^**2**^	ν(CH_3_)[(**D**)-*CH*_*3*_], ν_s_(CH_2_)[(**D**)-*CH*_*2*_-CH_2_-COOH]
1474	MV-MM^**3**^	ν(CH_3_)[(**Ring**)-*CH*_*3*_], ν(NH)_Ring_
1475	Pheophytin-a^**1**^	ν(CH_3_)[(**D**)-*CH*_*3*_], ν(CH_3_)[(**C**)-*CH*_*3*_], ω(CH)_**B−C**_
1475	py-Pheophorbide-a^**1**^	ν(CH_3_)[(**A**)-*CH*_*3*_], ν(CH_3_)[(**C**)-*CH*_*3*_], ν_s_(CH_2_)[(**D**)-*CH*_*2*_-CH_2_-COOH]
1479	DNChlC^**2**^	ρ(CH_2_)_**A−D**_, ν(C-C)_**C−D**_, ρ(CH_2_)[(**D**)-*CH*_*2*_-CH_2_-COOH]
1489	Chlorophyll-b^**1**^	ν(CH_3_)[(**A**)-*CH*_*3*_]
1490	DChlC-YTPP-1^**3**^	ν(C = C)_**C−D**_, ω(CH)[-C-*CH*-COOCH_3_], ν(CH_3_)[-C = CH-COO*CH*_*3*_]
1494	py-NChlC^**2**^	ν(C-N-C)_**D**_, ν_s_(CH_2_)[(**A**)-*CH*_*2*_-(**D**)], ρ(CH_2_)[(**D**)-*CH*_*2*_-CH_2_-COOH]
1495	C-E-RD^**3**^	ν(C = C)_**C**_, ν(CH_3_)[(**C**)-*CH*_*3*_], ω(NH)_**C**_, ω(CH)[(**C**)-*CH*O]
1501	Chlorophyllide-a^**1**^	ν(C = N)_**A**_, ν(CH_3_)[(**A**)-*CH*_*3*_], ν(C-C)[(**A**)-*C*H-*C*H_2_], ν_s_(CH_2_)[(**A**)-CH-*CH*_*2*_]
1501	Chlorophyll-b^**1**^	ν(C = N)_**A**_, ν(CH_3_)[(**D**)-*CH*_*3*_], ν(C-C)[(**A**)-*C*H-*C*H_2_], ν_s_(CH_2_)[(**A**)-CH-*CH*_*2*_]
1510	bc-NChlC^**3**^	ρ(CH_2_)[(**B**)-*CH*_*2*_-*CH*_*2*_-O-Glsy], ρ(CH_2_)[(**D**)-CH_2_-CH_2_-COO-*CH*_*2*_-Glsy]
1510	DChlC-YTPP-2^**3**^	ν(CH_3_)[(**D**)-*CH*_*3*_], ν(C-C)_**D**_, ρ(CH_2_)[(**D**)-*CH*_*2*_-CH_2_-COOH], ν(C = C)[-*C *= *C*H-COOH]
1531	pFChlC^**2**^	ν(CH_3_)[(**C**)-*CH*_*3*_], ν(C = C)_**C**_, ν(C-C)_**E**_
1535	C-E-RD^**3**^	ν(C-C)[(**C**)-*C*HO], ω(NH)_**C**_, ν(C = C)_**C**_, ν(CH_3_)[(**C**)-*CH*_*3*_]
1540	bc-FChlC^**3**^	ν(CH_3_)[(**C**)-*CH*_*3*_], ν(C = C)_**C**_, ν(C-N)_**C**_, ν(C-C)_**E**_
1541	Pheophorbide-a^**1**^	ν(C = C)_**D−E**_, ν(C = N)_**C**_, ν(C-C)_**B**_
1566	Chlorophyllide-a^**1**^	ν(C-C)_**A**_, ν(C-C)_**A−B**_
1567	Chlorophyll-a^**1**^	ν(C-C)_**A**_, ν(C-C)_**A−B**_
1572	Chlorophyll-b^**1**^	ν(C-C)_**A**_, γ(C-C)_**B**_
1587	Pheophorbide-a^**1**^	ν(C-N)_**C**_, ω(NH)_**C**_, ν(C = C)_**B=C**_, ν(C = C)_**B**_, ν(C = N)_**B**_
1611	Pheophytin-a^**1**^	ν(C-N)_**A**_, ω(NH)_**A**_, ν(C-C)_**A**_
1616	DPiChlC^**2**^	ν(C-N)_**D**_, ν(C = C)_**A−D**_
1619	bc-FChlC^**3**^	ν(C-N)_**D**_, ω(CH)_**A−D**_
1619	Chlorophyllide-a^**1**^	ν(C = C)_**B**_, ν(C = C)[**B** = *C*-**C**], ν(C = C)_**C**_
1619	PiChlC^**2**^	ν(C-N)_**D**_, ν(C = C)_**A−D**_, ν(C-N)_**C**_
1623	Chlorophyll-a^**1**^	ν(C = C)_**B−C**_, ν(C-N)_**B**_, ν(C = C)_**C**_
1629	Chlorophyll-b^**1**^	ν(C-C)_**D−E**_, ν(C = C)_**C**_, ν(C = C)_**D**_
1631	py-Pheophorbide-a^**1**^	ν(C = C)_**A−D**_, ν(C-C)_**C**_, ν(C = C)_**A−B**_

Rings are assigned a letter dependant on their relative position and whether the ring structure contains a nitrogen, i.e., using chlorophyll-b as an example, the top left ring, containing CH_3_ and CHCH_2_ groups, is assigned “A”; the top right ring containing CHO and CH_2_CH_3_ groups is assigned “B”; for the bottom right bi-ring structure, the right ring containing nitrogen and a CH_3_ group is assigned “C”, whilst the left ring containing a COOCH_3_ chain and a C = O is assigned “E”; and finally, the bottom left ring containing the CH_2_CH_2_COOPhytol chain is assigned “D”. This nomenclature is consistent for all catabolites except for: MV-MM and ME-MM where a single ring is present; C-E-RD, where only rings C and E are present; and ChlC-YTPP structures where the E ring has cleaved.

Most of the assignments are similar, with wagging of the C-H bonds between rings and on rings; wagging of the N-H bonds on rings; stretching and scissoring of CH_3_ groups on rings; stretching of C-C and C = C bonds on and between rings; stretches of C-N bonds on rings; and rocking or scissoring of CH_2_ groups following ring D being common. Spatially unique bond vibrations include the stretching of the CH_3_ group on the -C=CHCOOCH_3_ chain of DChlC-YTPP-1; the wagging of the C-H bond of the CHO group attached to ring C on C-E-RD; the rocking of the CH_2_ groups preceding glucosyl on the CH_2_CH_2_O-glucosyl chain attached to ring B of bc-NChlC; the rocking of the CH_2_ group preceding glucosyl on the CH_2_CH_2_COOCH_2_-glucosyl chain attached to ring D of bc-NChlC; the stretching of the C = C bond on the -C=CHCOOH chain of DChlC-YTPP-2; and finally, the stretching of the C-C bond on the ring C to CHO group on C-E-RD. All vibrating groups are highlighted in bold.

These spatially unique vibrations were found at: 1490, 1495, 1510 and 1535 cm^− 1^. Upon comparing to the experimental data, peaks were found at 1490 and 1495 cm^− 1^ in all senescence classes and 1510 cm^− 1^ in all senescence classes except SOL. A peak at 1535 cm^− 1^ was not present in any senescence class. As 1490, 1495 and 1510 cm^− 1^ are shared between more than one chlorophyll catabolite, specifically: chlorophyll-b and DChlC-YTPP-1; py-NChlC and C-E-RD; and bc-NChlC and DChlC-YTPP-2 respectively, these peak relationships need to be examined.

In the non-senescent and early senescence classes (i.e., HOL, MinSOL-H, ModSOL-H or MinSOL), the 1490 cm^− 1^ peak is unlikely to be attributed to DChlC-YTPP-1 as this is a late-stage catabolite but could be attributed to chlorophyll-b, the first step in the catabolism chain. As the leaves progress through to the later senescence classes (i.e., ModSOL or SOL), this peak can now be attributed to DChlC-YTPP-1, thus potentially indicating a progression through senescence.

In the non-senescent classes (i.e. HOL, MinSOL-H, ModSOL-H), the 1495 cm^− 1^ is unlikely to be attributed to the py-NChlC or C-E-RD simulated catabolites as they are mid- and quasi-mid-stage catabolites respectively, thus they are likely to be present in MinSOL, ModSOL and SOL spectra, and this is seen in the experimental data.

In the non-senescent and early senescence classes (i.e. HOL, MinSOL-H, ModSOL-H or MinSOL), the 1510 cm^− 1^ peak is also unlikely to be attributed to bc-NChlC or DChlC-YTPP-2 as they are late-stage catabolites; and conversely, in later senescence classes (i.e. ModSOL or SOL) they are likely to be produced and the peak is absent in SOL but present in ModSOL, potentially hinting at further breaking down of these catabolites.

### Spectral classifications

**Fig. 3:**
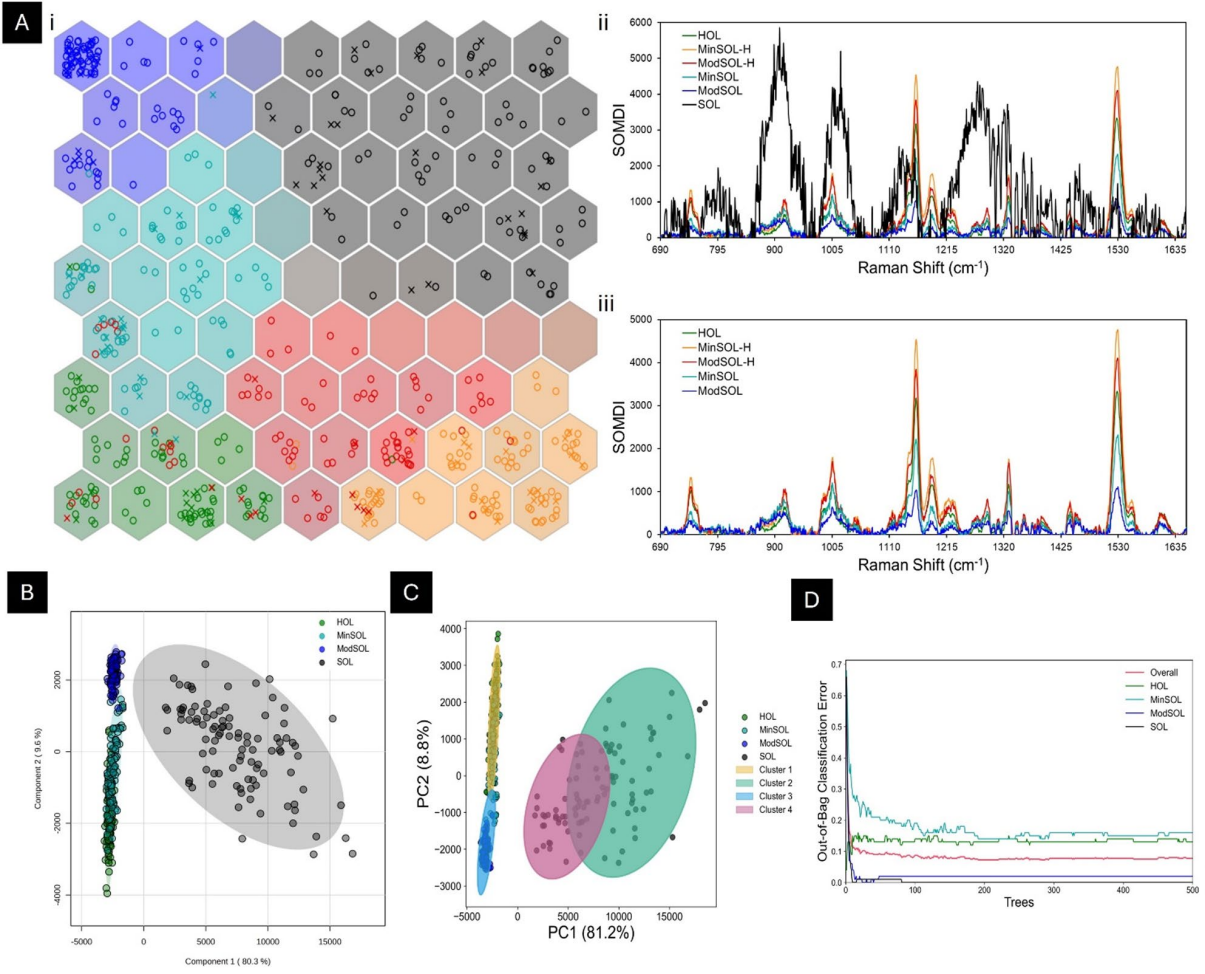
Classification Raman Spectroscopic Fingerprinting: **A** Clustering analysis of Raman spectra for healthy, minimally senesced, minimally senesced-healthy, moderately senesced, moderately senesced-healthy and fully senesced leaf tissue using (i) SOMs and SOMDIs both (ii) including and (iii) excluding fully senesced tissue spectra. **B** PLS-DA based on 5 component search and 5-fold cross validation method, with performance measured by Q^2^ = 0.87, R^2^ = 0.88 and overall accuracy = 0.74. **C** K-means clustering using principal components 1 and 2 with 4 clusters. **D **Random forest classification with 500 trees and 7 predictors. Overall, out-of-bag error was found to be 0.085. Individual class errors were found to be: HOL = 0.134, MinSOL = 0.177, ModSOL = 0.02 and SOL = 0.0

Through use of our in-house developed advanced artificial neural network, SKiNET, the spectral differences between HOL, MinSOL-H, ModSOL-H, MinSOL, ModSOL and SOL senescence classes can be revealed as an array of coloured hexagons. Figure 3A, i demonstrates all six senescence classes were successfully identified and the SOM appears to be well separated with distinct contiguous single-coloured regions pertaining to each respective senescence class with an overall training accuracy of: 95.51(± SD: 0.97%) and an overall testing accuracy of: 88.50(± 1.23%). The overall accuracy of the model was found to be 92.01%. Individually, training accuracies were: 87.93(± 4.21%), 95.94 (± 1.40%), 96.03 (± 2.03%), 95.51 (± 2.18%), 98.84 (± 0%) and 100 (± 0%) for HOL, MinSOL-H, ModSOL-H, MinSOL, ModSOL and SOL respectively. Individually, testing accuracies were: 68.18 (± 4.74%), 83.40 (± 1.92%), 78.15 (± 13.32%), 94.15 (± 1.76%), 99.20 (± 1.69%) and 100 (± 0%) for HOL, MinSOL-H, ModSOL-H, MinSOL, ModSOL and SOL, respectively.

Misclassifications occurred between HOL, MinSOL-H, ModSOL-H and MinSOL classes, shown by several mis-coloured training and testing spectra appearing in these regions (Fig. 3A, i). Whilst the spectra of these classes share similar morphology, especially in HOL, MinSOL-H and ModSOL-H tissue, these misclassifications were likely caused by subtle differences in peak distributions or structure. This in-turn leads to lower-than-expected testing accuracies for the HOL, MinSOL-H and ModSOL-H classes, contrary to MinSOL, ModSOL and SOL spectra where each demonstrated unique spectral morphologies.

The SOL-inclusive SOMDI (Fig. 3A, ii) highlights the importance of the 907 cm^− 1^ peak (associated with crystalline cellulose), the peaks at 1020 cm^− 1^ and 1525 cm^− 1^ (both associated with carotenoid-based bond vibrations), the 1157 cm^− 1^ peak (associated with carotenoid, chlorophyll-a and pheophytin-a-based bond vibrations), and the 1276 cm^− 1^ peak (associated with CH_3_-based bond vibrations) (Table 2); given in order of importance from most important to least, in separating the dataset into the regions seen within the SOM (Fig. 3A, i). This shows the heightened presence of crystalline cellulose and C-C coupled CH_3_ polyene rocking in the SOL tissue which drives the disparity between SOL tissue spectral morphology and other senescence class tissue morphologies, as indicated previously.

Following the observation of heightened crystalline cellulose in fully senesced tissue, the crystallinity of the cellulose in senescing leaf tissue was investigated using Eq. 1, which considers the relationship of the peaks at 1462 and 1481 cm^− 1^, associated with CH_2_-based bond vibrations in amorphous and crystalline cellulose respectively (Table 2).

*Equation* 1. Determination of Crystallinity using Raman Intensities, where I_1462_ and I_1481_ refer to the Raman intensity at 1462 and 1481 cm^− 1^ respectively [107].

1 $$\:{{X}_{c}}_{Raman}=\:\frac{{I}_{1481}}{{I}_{1462}+\:{I}_{1481}}$$

The crystallinity for HOL, MinSOL-H, ModSOL-H, MinSOL, ModSOL and SOL tissue was determined to be: 0.185 (± 0.207), 0.186 (± 0.189), 0.177 (± 0.191), 0.175 (± 0.222), 0.258 (± 0.226) and 0.594 (± 0.135) respectively (Supplementary Fig. 5). This indicates that as the leaf moves towards full senescence, the cellulose changes from an amorphous state to a crystalline one. All *post-hoc* Dunn’s test pairwise comparisons of Raman-derived crystallinity were not significantly different (*p* > 0.05) except for each pairing involving SOL tissue (for instance, HOL-SOL) where *p* < 0.001, highlighting the significant increase in crystallinity, supported by the increased brittleness of the leaf as it becomes fully senesced.

The SOMDI of the non-SOL classes was interrogated to identify peak importance for the separability of the remaining senescence classes (Fig. 3A, iii). This SOL-excluded SOMDI highlights the importance of the 1525, 1157, 1187 (associated with chlorophyll-a and lignin-based bond vibrations), 1003 (associated with carotenoid- and phenylalanine- based bond vibrations) and 1328 cm^− 1^ (associated with chlorophyll-a and pectin-based bond vibrations) peaks, given in order of importance from most important to least, in separating the dataset into the regions seen within the SOM (Fig. 3A, i). This indicates that the decrease of carotenoid, chlorophyll-a and pheophytin-a-based peaks are notable distinguishing markers between the respective classes (HOL, MinSOL, ModSOL, MinSOL-H and ModSOL-H). These observations further confirm that each of these molecules are degrading through autumnal senescence, as expected across the senescence classes.

Due to the spectral similarity between the HOL, MinSOL-H and ModSOL-H tissue, further classification methods were performed solely on HOL, MinSOL, ModSOL and SOL samples. For the PLS-DA (Fig. 3B), k-means clustering (Fig. 3C) and random forest (Fig. 3D) plots, the 700, 744, 854, 898, 917, 1003, 1048, 1147, 1157, 1226, 1288, 1309, 1328, 1343, 1355, 1451, 1456, 1494, 1525 and 1606 cm^− 1^ peaks were selected to represent a spectral sample. These peaks were primarily selected to represent common plant biomolecules, such as the 700 cm^− 1^ chlorophyll-a-related peak, the 898 cm^− 1^ cellulose, pectin and xylan-related peak, the 1147 cm^− 1^ chlorophyll-a and pheophytin-a-related peak and the 1525 cm^− 1^ carotenoid-related peak, for a full list of peak identities please see Table 2.

*PLS-DA*: PLS-DA further evidenced the disparity of the HOL, MinSOL, ModSOL and SOL class spectra, with a separation accuracy of 74% and performance (Q^2^) of 0.87 (Fig. 3B). Herein, SOL tissue is denoted by a single region and ModSOL is mostly denoted by a single cluster, whilst HOL and MinSOL tissue are represented by a singular mixed area with only the lower 95% confidence interval of the HOL tissue being isolated. This suggests that whilst the SOL and ModSOL spectra are distinguishable, the MinSOL and HOL spectra are mostly similar according to the PLS-DA model, in contrast to the SOMs and SOMDIs prior (Fig. 3A). However, the SOM-derived model included all spectral features and shifts, unlike the limited number the PLS-DA model was subjected to. This PLS-DA model biplot (Supplementary Fig. 6A), indicates the 1157 and 1525 cm^− 1^ carotenoid-related peaks were significant separators for the HOL and MinSOL spectra whilst the 898 cm^− 1^ cellulose, pectin and xylan-related peak was the most significant separator for the SOL spectra. Examining the variable importance in projection (VIP) score plot for the PLS-DA model (Supplementary Fig. 6B), we note the top five-most influential peaks driving separation observed in Fig. 3B. These are the 898, 917, 1288, 1525 and 1328 cm^− 1^ peaks from most to least influential representing: cellulose, pectins and xylan; cellulose and lignin; CH_2_ and CH_3_ groups; carotenoids; and chlorophyll-a and pectins, respectively (Table 2), thus highlighting potential changes to carotenoids, cellulose, chlorophyll-a and pectins as contributors to the changes in spectra morphology.

The SOL-excluded PLS-DA shows a separation accuracy of 87% and performance (Q^2^) of 0.808 (Supplementary Fig. 7) and shows a similarly mixed region between HOL and MinSOL spectra with a minimally mixed ModSOL region. The biplot further highlights the significance of the 1157 and 1525 cm^− 1^ carotenoid-related peaks as a separator for HOL tissue (Supplementary Fig. 8A). The VIP score indicates the top five most influential peaks leading to the separation are the: 1525, 1157, 1147, 1328 and 744 cm^− 1^ peaks from most to least influential representing: carotenoids; carotenoids; chlorophyll-a and pheophytin-a; chlorophyll-a and pectins; and chlorophyll-a and pectins, respectively (Table 2) (Supplementary Fig. 8B). This provides further evidence of carotenoids, chlorophyll-a and pectins being important discriminators between different stages of autumnal senescence.

*k-means Clustering*: Using k-means clustering, another partitional clustering method, (Fig. 3C), we find a similar trend to the PLS-DA model: the HOL and MinSOL regions overlap with some non-mixing at the regional extremes and ModSOL appearing mostly as a separate region. Unlike the PLS-DA plots (Fig. 3B) and the SOM (Fig. 3A, i), we see SOL data separating into two distinct regions. From this, 98/100 HOL spectra and 81/100 MinSOL spectra separate into cluster 1; 2/100 HOL spectra, 19/100 MinSOL spectra and all 100 of the ModSOL spectra separate into cluster 3; 52/100 SOL spectra separate into cluster 2; and finally, 48/100 SOL spectra separate into cluster 4 (Supplementary Fig. 9A).

Clusters 1 and 3, are morphologically alike with cluster 1 having the significantly higher intensity range (3571.92 ± 551.66 a.u. for cluster 1 and 1341.41 ± 518.55 a.u. for cluster 3, *p* < 0.001) (Supplementary Fig. 9B). This disparity suggests the HOL and MinSOL spectra in cluster 3 have a lower chlorophyll-a concentration, like that of ModSOL, given the aforementioned residual fluorescence of chlorophyll-a at 830 nm, further confirming a decrease of intensity as a key differentiator between HOL, MinSOL and ModSOL senescence classes.

Clusters 2 and 4 are also morphologically comparable (Supplementary Fig. 9C), however cluster 4 had a significantly greater overall intensity range (9942.69 ± 2224.98 a.u. for cluster 2 and 14493.02 ± 2345.05 a.u. for cluster 4, *p* < 0.001). This suggests there are additional biochemical changes observed within SOL tissue, as these changes cannot be fully explained by differing chlorophyll-a levels. It is possible that the samples from cluster 2 are closer to abscission and have less chlorophyll-a, such that fluorescent components within the mesophyll cells are subsequently more accessible, as they are no longer being spectrally quenched behind a more dominant chlorophyll-a Raman signal, and thus increasing the global intensity across the Raman spectra. From this, *a posteriori* the samples from Cluster 4 had a lower level of chlorophyll degradation or in Cluster 4 there is more unexplained fluorescence or Raman-active bonds, possibly due to the samples from Cluster 4 being closer to abscission.

*Random Forest Classification*: Using random forest classification, a supervised machine learning algorithm, we can further validate prior findings whilst probing differences between senescence classes (Fig. 3D). Whilst the ModSOL and SOL classes tended to separate the best (< 30 trees), akin to the SOM findings, the HOL and MinSOL classes were still fluctuating at 500 trees, indicating difficulties in separating these classes, likely due to overlapping features. The out-of-bag error was 7.75%, indicating a 92.25% accuracy, with class-specific accuracies being: 87%, 84%, 98% and 100% for HOL, MinSOL, ModSOL and SOL classes respectively. By examining which peaks would drive the greatest loss of mean accuracy if excluded from the dataset, the 1157, 917, 898, 1525 and 1288 cm^− 1^ peaks were found to be the top five most influential peaks for successful classification (Supplementary Fig. 10). These peaks reflect bond vibrations associated with carotenoids, chlorophyll-a and pheophytin-a; cellulose and lignin; cellulose, pectins and xylan; carotenoids; and aliphatics, respectively, thus providing further evidence that these molecules are important for distinguishing between senescence classes, previously confirmed in the other analysis methods used herein. One advantage of a random forest classifier compared to SKiNET, is the ability to identify outliers within the dataset (Supplementary Fig. 11). The top five outliers were determined to be ModSOL-62, SOL-88, ModSOL-100, SOL-80 and SOL-75. These outliers were similar in spectral morphology to the remainder of the class but had greater amounts of spectral noise, leading to loss of peak definition either across the entire wavenumber range or in specific spectral regions, especially the 1215–1350 cm^− 1^ region for SOL samples, potentially illustrating differences to the pectin biochemistry across these samples.

### Spectral investigations

*Spectrophotometry*: To further explore changes to chlorophyll-a through the senescence classes, spectrophotometry at 600 nm wavelength was conducted due to the proximity of a broad peak attributable to chlorophyll-a (Supplementary Fig. 12). Sample absorbance significantly decreased from 1.67 (± 0.0416) to 0.298 (± 0.00764) a.u. from HOL to SOL respectively (p = 0.0134), thus confirming a significant decrease in chlorophyll concentration. This relationship follows a negative exponential trend (Supplementary Fig. 12), explained by the nature of the loss of chlorophyll, as at each senescence class from HOL to MinSOL to ModSOL to SOL, there is less chlorophyll-a to degrade at each senescence stage. Whilst the spectrophotometric data confirms a loss of chlorophyll-a, the average spectral area over the whole spectral range, determined using Simpson’s rule, follows a strong positive correlation between the loss of absorbance and the loss of area (R^2^ = 0.925) from HOL to ModSOL tissue and weakly positive (R^2^ = 0.179) from HOL to SOL (Supplementary Fig. 13). The SOL-exclusive correlation confirms a loss of Raman intensity mirroring the loss of chlorophyll-a in the sample. The greater area-under-the-curve in SOL tissue, however, indicates a slew of newly present Raman active molecules present, confirming earlier suspicions. This warrants additional metabolite screening to determine the formation of new or intermediary compounds.

**Fig. 4:**
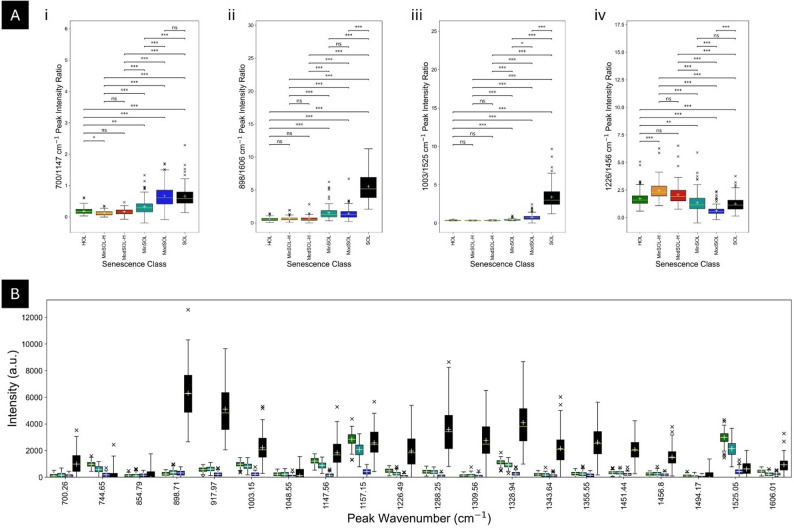
Processing Analysis of Raman Spectroscopic Fingerprinting.** A** Ratio analysis using box and whisker plots of the (i) 700/1147 cm^-1^, (ii) 898/1606 cm^-1^, (iii) 1003/1525 cm^-1^ and (iv) 1226/1456 cm^-1^ Raman peak intensity ratios. Brackets denote significantly different pairs of peak intensity ratios compared via a Kruskal-Wallis ANOVA followed up with a Dunn’s post-hoc test with ns > 0.05, *p < 0.05, **p < 0.01 and ***p < 0.005. **B** Distribution analysis of peaks-of-interest Raman intensity using box and whisker plots

*Peak ratios*: Another method of interrogating senescence class spectral differences is to probe the peak ratios between spectral features associated with common plant biomolecules. As such, the 700/1147 cm− 1 (Fig. 4A, i), 898/1606 cm− 1 (Fig. 4A, ii), 1003/1525 cm− 1 (Fig. 4A, iii) and 1226/1456 cm− 1 (Fig. 4A, iv) peak ratios representing the ratios associated with chlorophyll-a behaviour; the carbohydrate-lignin relationship; phenylalanine-carotenoid relationship; and the chlorophyll-a-α-amyrin relationship in the leaves through different stages of senescence respectively.

The 700/1147 cm^− 1^ peak ratio (Fig. 4A, i) ranges from 0.121 (± 0.0726) to 0.674 (± 0.384) from MinSOL-H to ModSOL tissue, following a linear relationship (R^2^ = 0.873) (Supplementary Fig. 14A). This suggests that chlorophyll is degrading at a consistent rate, further confirmed by correlating this ratio against spectrophotometric absorbance, where an exponential R^2^ of 0.980 is noted (Supplementary Fig. 15). This ratio-spectrophotometric absorbance relationship describes a decrease in absorbance, i.e., a decrease in chlorophyll-a, leading to an increase in ratio which may be caused by numerous factors. Given that the 1147 cm^− 1^ peak is associated with chlorophyll-a and pheophytin-a whereas the 700 cm^− 1^ peak is exclusively associated with chlorophyll-a, the intensity of the 1147 cm^− 1^ peak is expected to decrease more readily than the intensity of the 700 cm^− 1^ peak as the leaves move between senescence classes. Thus, a gradual increase in the ratio from HOL to MinSOL and a more pronounced increase from MinSOL to ModSOL tissue is seen. All *post-hoc* Dunn’s test pairwise comparisons were statistically different (*p* < 0.001) except for: HOL-MinSOL-H (*p* = 0.0387), HOL-MinSOL (*p* = 0.00121), and HOL-ModSOL-H, MinSOL-H-ModSOL-H and ModSOL-SOL (*p* > 0.05). These highlight minimal differences to the chlorophyll levels between HOL and MinSOL-H and between HOL and MinSOL tissue and similarities in the chlorophyll levels between HOL and ModSOL-H; MinSOL-H and ModSOL-H; and ModSOL and SOL tissue.

The 898/1606 cm^− 1^ peak ratio (Fig. 4A, ii) ranges from 0.534 (± 0.263) to 5.52 (± 1.92) from HOL to SOL tissue and appears to follow an exponential trend (R^2^ = 0.908) (Supplementary Fig. 14B). All *post-hoc* Dunn’s test pairwise comparisons were all significantly different (*p* < 0.001) except for: HOL-MinSOL-H, HOL-ModSOL-H, MinSOL-H-ModSOL-H, and MinSOL-ModSOL (*p* > 0.05). This further highlights the similarity in the carbohydrate-lignin relationship between the non-senescent class and the non-senescent subclasses and between minimally senesced and moderately senesced tissue whilst indicating a non-carbohydrate and non-lignin bond vibration contributor to either peak wavenumber. The 898 cm^− 1^ peak was additionally linked to: the early-stage pFChlC; the mid-stage DYChlC; and the late-stage ChlC-YTPPs and DChlC-YTPPs. The 1606 cm^− 1^ peak was additionally linked to: the early-stage pheophorbide-a; the mid-stage YChlC; the quasi-mid stage C-E ring derivative; and the late-stage ChlC-YTPP-1 and DChlC-YTPP-2. The 898 cm^− 1^ has two additional late-stage ChlCs, likely explaining the sharp increase seen in SOL tissue.

The 1003/1525 cm^− 1^ peak ratio (Fig. 4A, iii), ranges from 0.338 (± 0.0294) to 3.40 (± 1.52) from MinSOL-H to SOL tissue, producing an exponential relationship (R^2^ = 0.926) (Supplementary Fig. 14C). All *post-hoc* Dunn’s test pairwise comparisons were all significantly different at the *p* < 0.001 level except for: HOL-MinSOL-H, HOL-ModSOL-H and MinSOL-H-ModSOL-H (*p* > 0.05) and MinSOL-ModSOL (*p* = 0.0111), like the 898/1606 cm^− 1^ peak ratio. This further highlights the similarity in the phenylalanine-carotenoid relationship between the non-senescent tissue and non-senescent subclass tissue whilst confirming a progression of the ratio from these non-senescent classes to fully senesced tissue, suggesting a non-carotenoid bond and non-phenylalanine vibration contributor to either peak wavenumber. After interrogating the simulated Raman spectra, the mid-stage: DNChlCs, DYChlCs and DPiChlCs and the quasi-mid-stage methylethyl maleimide had a peak at 1003 cm^− 1^ whilst the mid-stage: RChlCs, YChlCs, DNChlCs, py-RChlCs and py-YChlCs had a peak at 1525 cm^− 1^. The lack of late-stage catabolite at 1003 cm^− 1^ (as the quasi-mid-stage catabolite further breaks down) or 1525 cm^− 1^ calls into question the exponential behaviour, however, an increase in the bonds associated with phenylalanine could explain this observation.

The 1226/1456 cm^− 1^ peak ratio (Fig. 4A, iv), ranges from 0.627 (± 0.483) to 2.46 (± 0.829) from ModSOL to MinSOL-H tissue with a weak exponential relationship (R^2^ = 0.435) (Supplementary Fig. 14D). The behaviour of this ratio cannot, at present, be fully explained solely by the degradation of chlorophyll. However, in the SOL spectra, as previously discussed, there is an unexplained increase of overall intensity in the 1215–1320 cm^− 1^ peak region, potentially contributing to the relative increase of the ratio from ModSOL to SOL tissue. All *post-hoc* Dunn’s test pairwise comparisons were all significantly different (*p* < 0.001) except for: HOL-ModSOL-H, MinSOL-H-ModSOL-H and MinSOL-SOL, (*p* > 0.05), and HOL-MinSOL (*p* = 0.00822), further highlighting dissimilarity in the chlorophyll-a-α-amyrin relationship through all classes, especially in the senescent classes from MinSOL through to SOL, and also indicating non-chlorophyll-a and non-α-amyrin bond contributors to the peak wavenumbers. From the simulation data, a slew of mid-stage ChlCs have been identified at 1456 cm^− 1^ including: the mid-stage YChlCs, DNChlCs and DYChlCs, py-YChlCs; the quasi-mid-stage methylethyl maleimide and the late-stage, bc-NChlC indicating these could be contributing to the increase of the 1456 cm^− 1^ peak intensity. The late-stage DPiChlCs and ChlC-YTPP-1 were identified to be contributors to the 1226 cm^− 1^ peak, hence potentially raising the intensity of this peak in SOL compared to ModSOL.

*Box-and-Whisker distribution analysis*: Peak-specific relationships were examined using box-and-whisker plots of Raman intensities across each of the 20 chosen peaks-of-interest through each senescence class (Fig. 4B), resulting in four distinct patterns.

Firstly, the 700, 898, 917, 1048, 1288, 1309, 1343, 1355, 1451 and 1456 cm^− 1^ peak intensities appear to be similarly under expressed in HOL, MinSOL and ModSOL tissue compared to SOL tissue. These peaks represent bond vibrations associated with aliphatic compounds, carotenoids, cellulose, lignin, pectin, xylan and α-amyrin-associated with cuticular wax (Table 2). Whilst it is expected that some of these contributors either change or degrade, like the carotenoids, we see a sharp increase to the relative peak intensity in SOL tissue, suggesting an as-yet unknown Raman-active contributor or contributors to the regions in which these peaks lie and warrants further investigation.

The 744, 1494 and 1525 cm^− 1^ peak intensities progress from most over expressed in HOL tissue to most under expressed in SOL tissue, except 1525 cm^− 1^ where ModSOL is the most under expressed. These peaks represent bond vibrations associated with: chlorophyll-a and pectins, pheophytin-a, and carotenoids, respectively (Table 2). The progressive decrease to the 744 and 1494 cm^− 1^ peak intensities can be explained by chlorophyll-a and pheophytin-a degrading, however, carotenoid degradation would lead to a lower SOL intensity value of the 1525 cm^− 1^ peak compared to the ModSOL intensity. Hence, the increase in SOL intensity of the 1525 cm^− 1^ peak and the surrounding region warrant further investigation.

The 1003, 1147, 1157, 1226, 1328 and 1606 cm^− 1^ peak intensities decrease from HOL to ModSOL and then increase to a higher-than-HOL intensity in SOL tissue. These peaks represent bond vibrations associated with: carotenoids, chlorophyll-a, lignin, pectins, phenylalanine and pheophytin-a (Table 2). The decrease from HOL to ModSOL can be explained by the degradation of carotenoids, chlorophyll-a and pheophytin-a, the overexpression in SOL tissue cannot be accounted for. The most likely explanation is bond vibration contributors in this region from newly produced or more visible Raman-active molecules leading to an increase in relative intensity across these wavenumbers.

Finally, the 854 cm^− 1^ peak, associated with pectins (Table 2), moves from a similar intensity value in HOL and MinSOL, to a higher value in ModSOL, to a lower-than-HOL value in SOL tissue. All *post-hoc* Dunn’s test pairwise comparisons for this peak reveal statistically non-significant differences (*p* > 0.05) except for the ModSOL-SOL pair (*p* = 0.00228). One possible explanation is a mid-stage ChlC with bond vibrations in this region are being produced as the leaf moves from HOL to MinSOL to ModSOL tissue which then subsequently degrades from ModSOL to SOL tissue, leaving a relative intensity which is significantly lower than ModSOL tissue. Another explanation is an adjustment of pectin structure from α- to β- anomeric form within the cell walls through senescence, as the 854 cm^− 1^ peak is associated with the α-anomeric form whereas the 898 cm^− 1^ peak is associated with the β-anomeric form and a decrease of the 854 cm^− 1^ peak is accompanied by an increase to the 898 cm^− 1^ peak [108, 109]. Whilst the precise content of the *Q. robur* pectin content in non-venous tissue cell walls was not determined herein, it is known that pectins have widely varied functions [55]. Hence, an additional study investigating this anomeric shift in pectin structure is necessitated to further understand the role in autumnal senescence as the leaf approaches abscission.

### Summary

Overall, using Raman spectroscopy to elucidate the breakdown pattern of chlorophyll in *Quercus robur* has yielded promising results. Whilst no morphological change to the mesophyllic cell structure using confocal and optical microscopy was seen, respective colour changes observed using pixel and visual analyses infer a chemical change. The experimental Raman spectra revealed similarities in spectral morphology across non-senescent leaves and non-senescing areas present on minimally and moderately senescing leaves, however, intensity ranges did significantly differ, likely due to chlorophyll-a levels in the leaves measured. As leaves progress from non-senescent through to full senescence, the spectral morphology remains similar from non-senescent to minimally senesced tissue, then changes to non-major peak regions are observed in moderately senesced tissue before new major peaks and regions of interest are identified in fully senesced tissue, shown by integrating under-the-curve through Simpson’s rule. This analysis revealed differences between non-senescing leaves and non-senescent areas on minimally and moderately senescing leaves, likely due to the presence of an unidentified global indicator of senescence and confirm the appearance of new Raman-active bond-contributors in minimally, moderately and fully senesced tissue. To investigate these new Raman-active bond-contributors, computational analysis of expected and predicted chlorophyll catabolites was undertaken, where several unique bond vibrations were identified at 1490, 1495, 1510 and 1535 cm^− 1^;1490 and 1495 cm^− 1^ were in all senescence class experimental spectra and 1510 cm^− 1^ was present in all senescence class experimental spectra. These unique bond vibrations belong to mid, quasi-mid and late-stage chlorophyll catabolites showing a progression through the catabolites and potentially further degradation from late-stage products. Further investigations into spectra differences using SKiNET, PLS-DA, k-means and random forest model classification identified crystalline cellulose as an important class-specific differentiator and following analysis showed an increase in the crystallinity of cellulose as the leaf progresses through senescence. Other identified differentiators include: the 1157 and 1525 cm^− 1^ carotenoid peaks; the 898 cm^− 1^ cellulose, pectin and xylan peak; the 917 cm^− 1^ chlorophyll-a and pheophytin-a peak; and the 1288 cm^− 1^ aliphatic peak. K-means clustering resulted in the senesced spectra being separated into two clusters, likely different due to leaf samples being closer to abscission. Two other clusters containing mostly moderately senesced spectra (with some non-senescing and minimally senesced spectra) and the other mostly containing non-senescing and minimally senesced spectra were identified, likely different due to chlorophyll levels changing between samples. Spectrophotometry at 600 nm confirmed the loss of chlorophyll-a but when combined with area-under-the-curve using Simpson’s rule showed an unexplained heightened intensity in fully senesced tissue, further confirming newly present Raman-active bond contributors in fully senesced tissue. Peak ratio analysis further confirmed the presence of newly present Raman-active bond contributors as the 700/1147 and 1003/1525 cm^− 1^ peak ratios indicating chlorophyll-a behaviour and phenylalanine-carotenoid relationship respectively show an unexplained exponential increase given both chlorophyll-a and carotenoid levels are expected to decrease similarly in *Quercus robur*. The 898/1606 cm^− 1^ peak ratio indicating carbohydrate-lignin relationship also shows an exponential increase associated with either an increased presence of chlorophyll catabolites or the changing of pectin from the α- to β- anomeric form. The 1226/1456 cm^− 1^ peak ratio associated with chlorophyll-a and α-amyrin indicated a complex relationship and a slew of mid- and late- stage chlorophyll catabolites were identified at these wavenumbers from the simulated data. Finally, analysis of the peaks-of-interest further indicated unidentified Raman-active bond contributors across all regions of fully senesced spectra whilst further suggesting the changing of pectin from the α- to β- anomeric form and the cellulose becoming more crystalline through autumnal senescence.

## Conclusions

Herein, we have explored autumnal senescence-induced biochemical changes to non-venous leaf tissue from *Quercus robur* as the leaf progresses from non-senescent through to fully senesced. Experimental Raman spectroscopy at 830 nm combined with simulated Raman spectroscopy with density-functional theory calculations, spectrophotometry at 600 nm, colour channel analysis and use of an advanced artificial neural network were used to identify evidence of the presence of complex mid-, quasi-mid- and late-stage chlorophyll catabolites and any other identifying marker of the progression of senescence from non-senesced leaf tissue through to minimally, moderately and fully senesced tissue.

The use of the SKiNET algorithm provided testing accuracies of 68.18% for non-senescent tissue, 83.40% for visually non-senescing tissue on minimally senesced leaves, 78.15% for visually non-senescing tissue on moderately senesced leaves, 94.15% for minimally senesced tissue, 99.20% for moderately senesced tissue and 100% for fully senesced tissue. Further analysis of the self-organising map discriminant index highlighted the importance of crystalline cellulose to the separation between these senescence classes, and we were able to demonstrate, an increase of Raman-derived crystallinity (from 0.185 in non-senescent tissue to 0.594 in fully senesced tissue) as the leaf approaches full senescence, explaining in-part the increase of brittleness of the leaves pre-abscission. Analysing the ratio of the peak intensities at 1226 and 1456 cm^− 1^ relating to chlorophyll-a-α-amyrin behaviour indicated a complex relationship which needs to be investigated further. Analysing the ratio of the intensities at 700 and 1147 cm^− 1^ relating to chlorophyll-a behaviour; 1003 and 1525 cm^− 1^ relating to the phenylalanine-carotenoid relationship and 898 and 1606 cm^− 1^ relating to carbohydrate-lignin behaviour indicated the presence of new Raman-active bond contributors and the latter ratio also indicated the changing of pectin from the α- to β- anomeric form. A decrease in the intensity of the 854 cm^− 1^ peak and coincident increase in the intensity of the 898 cm^− 1^ peak further suggests this α- to β- anomeric form change of pectin, which needs to be investigated further. Furthermore, in fully senesced tissue spectra there appears to be an unexplained increase of overall intensity in the 1215–1320 cm^− 1^ peak region, further highlighting the presence of new Raman-active bond contributors. Finally, through computational analysis of expected and predicted chlorophyll catabolites, several unique bond vibrations were identified at 1490, 1495, 1510 and 1535 cm^− 1^; 1490 and 1495 cm^− 1^ were found in all senescence classes; 1510 cm^− 1^ was found in all senescence classes except in fully senesced tissue; and 1535 cm^− 1^ was not found in any collected spectra. These unique bond vibrations belong to mid, quasi-mid and late-stage chlorophyll catabolites showing a progression through the catabolites and potentially further degradation from late-stage products.

Thus, whilst the full process of foliaceous ageing remains elusive and largely enigmatic, we have provided insight into the biomolecular process of senescence. By developing a classification model driven by advanced machine learning and AI-integration with Raman spectroscopy, we demonstrated the most dissimilar spectral peaks between senescence classes. Thus, we provide the first platform to accurately determine the relative progressive position of a leaf through its lifecycle, whilst simultaneously demonstrating the complexities of autumnal senescence not just through the breakdown of chlorophyll but through the changes to carbohydrates, especially cellulose and pectin, as deciduous leaves approach abscission.

The ability to determine the progressive position of a leaf through its lifecycle has applications outside of autumnal senescence, most notably in pathogen-specific senescence observed through disease as early indicators of a pathogen’s presence on the leaf could be from these senescence-induced biochemical changes within the leaf. Additionally, this has significant industrial applications, including notably, in pharmaceuticals, medicinal chemistry and biotechnology, where frequently high value compounds are extracted either for pharmacological or cosmetic exploitation. Specifically, some of these drug classes include steroids, local anaesthetics, analgesics, antitumour agents and cardiac glycosides (e.g., digoxin). Similarly, chlorophyll has several applications outside of the photosynthetic activity most associated, including roles in food colourings (characteristic greening of edible products) and additives (e.g., chlorophyllin) for suggested antioxidants in foods.

## Supplementary Information

Supplementary Material 1.

## Data Availability

The datasets used throughout this study are available from the corresponding author upon reasonable request.
