# Supplementary material for: Multimodal machine learning and Raman spectroscopy uncover biochemical pathways of autumnal leaf senescence
Source: BMC Plant Biol. 2026 Feb 17;26:541. doi: 10.1186/s12870-026-08369-1 (PMC13014873; doi:10.1186/s12870-026-08369-1)
Supplement: Supplementary file 1 — Supplementary Material 1. [file 12870_2026_8369_MOESM1_ESM.docx]

**---Supplementary Information---**

**Hybrid Machine Learning and Raman Spectroscopic Analysis of Ageing Leaves. A Biochemical Analysis and Characterisation of Autumnal Senescence**

*Kieran R. Clark, Jarrod L. Thomas and Pola Goldberg Oppenheimer*

**Contents – Tables and Figures**

[**Table S1: Expanded bond vibration profiles and tentative assignments for spectral classes.** §, Δ, σ and ø refer to the presence of the bond vibration in non-senesced, minimally senesced, moderately senesced and fully senesced leaf tissue, respectively. For bond vibration notation, see **Table 2** in the main text. 3](#_Toc218680041)

[**Figure S1: Individual Leaf Class Senescence Class Spectra.** Molecular profiling using Raman spectroscopic fingerprinting of **(A)** healthy [HOL], **(B)** minimally senescence [MinSOL], **(C)** minimally senescence-healthy [MinSOL-H], **(D)** moderately senesced [ModSOL], **(E)** moderately senesced-healthy [ModSOL-H] and **(F)** fully senesced [SOL] tissue……………………………………………………………………………………………………….5](#_Toc218680114)

[**Figure S2:** **Identified Regions of Interest in Spectral Profiles. (A)** 860-970 cm^-1^. **(B)** 980-1055 cm^-1^. **(C)** 1095-1170 cm^-1^. **(D)** 1205-1335 cm^-1^ for non-senescent [HOL] **(i)**, minimally senesced-healthy [MinSOL-H] **(ii)**, moderately senesced-healthy [ModSOL-H] **(iii)**, minimally senesced [MinSOL] **(iv)**, moderately senesced [ModSOL] **(v)** and fully senesced [SOL] **(vi)** tissue. 6](#_Toc218680115)

[**Figure S3:** **Area-Under-the-Curve for Identified Regions of Interest in Spectral Profiles. (A)** 860-970 cm^-1^. **(B)** 980-1055 cm^-1^. **(C)** 1095-1170 cm^-1^. **(D)** 1205-1335 cm^-1^ for non-senescent [HOL] **(i)**, minimally senesced-healthy [MinSOL-H] **(ii)**, moderately senesced-healthy [ModSOL-H] **(iii)**, minimally senesced [MinSOL] **(iv)**, moderately senesced [ModSOL] **(v)** and fully senesced [SOL] **(vi)** tissue. Brackets represent significantly different pairs of region area-under-the-curves compared via a Kruskal-Wallis ANOVA followed up with a Dunn’s *post-hoc* test with ns>0.05, **p<0.01 and ***p<0.005. 7](#_Toc218680116)

[**Figure S4: Simulated Raman Spectra Mechanism of Chlorophyll Catabolism.** MV-MM: methylvinyl maleimide; ME-MM: methylethyl maleimide; C-E-RD: C-E ring derivative; HTA: hematinic acid. Abbreviations starting with D: dioxobilin-type compounds, abbreviations starting with py-: pyro compounds post-decarboxylation of the methyl ester functional group and abbreviations starting with bc-: bicyclic chlorophyll catabolites. For all other abbreviations, see text. 8](#_Toc218680117)

[**Figure S5: Raman-Derived Crystallinity Plot.** Crystallinity of non-senescent [HOL], minimally senesced-healthy [MinSOL-H], moderately senesced-healthy [ModSOL-H], minimally senesced [MinSOL], moderately senesced [ModSOL] and fully senesced [SOL] leaf tissue determined using the intensities of the 1462 and 1481 cm^-1^ peaks, as described by Schenzel, Fischer and Brendler [30]. Brackets denote significantly different pairs of Raman-derived crystallinities compared via a Kruskal-Wallis ANOVA followed up with a Dunn’s post-hoc test with ns>0.05 and ***p<0.005. 9](#_Toc218680118)

[**Figure S6: SOL-Included PLS-DA Biplot and Variable Importance Plots.** The SOL-included PLS-DA biplot **(A)**, performance measured by Q^2^ = 0.87, R^2^ = 0.88 and overall accuracy = 0.74, and variable importance plot **(B)**. 10](#_Toc218680119)

[**Figure S7: SOL-Excluded PLS-DA.** SOL-excluded PLS-DA based on 5 component search and 5-fold cross validation method with performance measured by Q^2^ = 0.81, R^2^ = 0.83 and overall accuracy = 0.87**.** 11](#_Toc218680120)

[**Figure S8: SOL-Excluded PLS-DA Biplot and Variable Importance Plots.** The SOL-excluded PLS-DA biplot, with performance measured by Q^2^ = 0.81, R^2^ = 0.83 and overall accuracy = 0.87 **(A)** and variable importance plot **(B)**. 12](#_Toc218680121)

[**Figure S9: K-means Cluster Analysis.** The stacked plot for the overall averages of Clusters 1-4 **(A)** with individual stacked plots for Clusters 1 and 3 **(B)** and Clusters 2 and 4 **(C)** 13](#_Toc218680122)

[**Figure S10:** **Random Forest Analysis.** The variable importance plot for the random forest model **(A)** with individual violin plots showing the distribution of peak intensity (a.u.) of the top-5 peaks: 1157 **(B)**, 917 **(C)**, 898 **(D)**, 1525 **(E)** and 1288 cm^-1^ **(F)** for healthy (HOL), minimally senesced (Min-Sen, MinSOL), moderately senesced (Mod-Sen, ModSOL) and fully senesced (Sen, SOL) oak leaf tissue**.** 14](#_Toc218680123)

[**Figure S11**: **Random Forest Outlier Analysis.** The overall outlier plot **(A)** and Raman spectra comparing sample ModSOL-62 against ModSOL average **(B)**; sample SOL-88 against SOL average **(C)**; sample ModSOL-100 against ModSOL average **(D)**; sample SOL-80 against SOL average **(E)**; and sample SOL-75 against SOL average **(F)**. Samples are shown as blue, averages as orange. 15](#_Toc218680124)

[**Figure S12:** **Spectrophotometric Analysis and Trend.** Spectrophotometric absorbance measured at 600 nm for non-senescent [HOL], minimally senesced [MinSOL], moderately senesced [ModSOL] and fully senesced [SOL] leaves. HOL-SOL (p=0.0134) was denoted as the sole significant differentiator between datasets. Remaining classes were devoid of any statistical significance. Bar chart presented with ±SD and with exponential line-of-best-fit (R^2^=0.994). 16](#_Toc218680125)

[**Figure S13:** **Area-Under-the-Curve-Spectrophotometric Absorbance Trend Analysis.** Correlation of whole spectral range area-under-the-curve against spectrophotometric absorbance measured at 600 nm for non-senescent [Green - HOL], minimally senesced [Teal - MinSOL], moderately senesced [Blue - ModSOL] and fully senesced [Black - SOL] tissue. Scatter chart presented with ±SD and with linear line-of-best-fit, SOL-excluded R^2^=0.925, SOL-included R^2^=0.179. 17](#_Toc218680126)

[**Figure S14.** **Peak Intensity Ratio Distribution and Trends.** Ratio analysis using bar charts of the (**A**) 700/1147 cm^-1^, (**B**) 898/1606 cm^-1^, (**C**) 1003/1525 cm^-1^ and (**D**) 1226/1456 cm^-1^ Raman peak intensity ratios each with lines of best fit showing R^2^ values of: 0.873, 0.908, 0.926 and 0.435 respectively. 18](#_Toc218680127)

[**Figure S15: 700/1147 cm-^1^ Peak Intensity Ratio-Spectrophotometric Absorbance Trend Analysis.** Correlation of 700/1147 cm^-1^ peak intensity ratio against spectrophotometric absorbance measured at 600 nm for non-senescent [Green - HOL], minimally senesced [Teal - MinSOL], moderately senesced [Blue - ModSOL] and fully senesced [Black - SOL] tissue. Scatter chart presented with ±SD and with exponential line-of-best-fit, R^2^=0.980. 19](#_Toc218680128)

**Table S1: Expanded bond vibration profiles and tentative assignments for spectral classes.** §, Δ, σ and ø refer to the presence of the bond vibration in non-senesced, minimally senesced, moderately senesced and fully senesced leaf tissue, respectively. For bond vibration notation, see **Table 2** in the main text.

| Raman Shift (cm^-1^) | Bond Vibration | Tentative Assignment | Senescence Class | Reference |
| --- | --- | --- | --- | --- |
| 700 | Not Identified | Chlorophyll-a | §, Δ, σ, ø | [1] |
| 715 | γ(C–OH)_ring_ | Pectins | σ, ø | [2] |
| 744 | δ(N–C–C)  γ(C–OH)_COOH_ | Chlorophyll-a  Pectins | §, Δ, σ | [3, 4]  [2, 5] |
| 752 | Not Identified | Xyloglucan | §, Δ | [6] |
| 793 | γ(C–OH)_COOH_ | Pectins | §, σ, ø | [2] |
| 806 | δ(C–C–O)_endocyclic_ | D-ribose | σ, ø | [7] |
| 830 | γ(C–OH)_COOH_ | Pectins | §, Δ, σ, ø | [2] |
| 834 | γ(C–OH)_ring_ | Pectins | ø | [2] |
| 854 | C–C–O–C–O skeleton | Pectins | §, Δ, σ, ø | [2, 8] |
| 866 | C–O–C_skeleton_ | Carbohydrates | §, Δ | [9] |
| 873 | ν(C–C) | D-ribose | §, Δ, σ, ø | [7] |
| 879 | ν(C–C) | D-ribose | ø | [7] |
| 890 | δ(C–C–H), δ(C–OH) | Pectins | Δ, σ | [2] |
| 898 | H–C–C, H–CO bending  δ(C–C–H), δ(C–OH)  δ(CH)_aromatic_ | Cellulose (amorphous)  Pectins  Xylan | §, Δ, σ, ø | [10, 11] [2] [12] |
| 900 | ν(C–O–C)_in-plane, symm_ | Not Identified | σ | [13] |
| 903 | δ(C–C–H)_exocyclic_ | 2-deoxy-D-erythropentose | Δ, σ, ø | [7] |
| 907 | H–C–C, H–CO bending | Cellulose (crystalline) | §, Δ, σ, ø | [11] |
| 917 | Not Identified  ν(C–O–C)_symm_ | Cellulose  Lignin | §, Δ, σ, ø | [5, 13] |
| 936 | Unknown | D-ribose | Δ, σ, ø | [7] |
| 942 | ν(C–C) | D-ribose | §, Δ, σ | [7] |
| 951 | δ(C–C–H), δ(C–OH) | Pectins | Δ, σ, ø | [2] |
| 964 | ν(C–H)_out-of-plane_ | Carotenoids | §, Δ | [14] |
| 990 | ν(C–C) | D-ribose | §, Δ, σ, ø | [7] |
| 996 | ρ(CH_2_) | Not Identified | Δ, σ | [13] |
| 1003 | ρ(CH_3_)_polyene_ | Carotenoids | §, Δ, σ, ø | [5, 15] |
|  | ν(C–C) | Phenylalanine |  | [16] |
| 1016 | δ(C–OH) | D-ribose | §, Δ | [7] |
| 1020 | C–C-coupled ρ(CH_3_)_polyene_ | Carotenoids | §, Δ, σ, ø | [17, 18] |
| 1032 | ν(CC)(CO) | Pectins | Δ, σ, ø | [2] |
| 1040 | ν(CO) | 1º Alcohol | σ | [13] |
| 1048 | ν(C–O), ν(C–C),  δ(C–OH) | Cellulose | §, Δ, σ, ø | [5, 19] |
|  | ν(CC)(CO) | Pectins |  | [2] |
| 1063 | ν(CO) | 2º Alcohol | §, Δ, σ | [13] |
| 1079 | ν(CO), δ(OH) | Pectins | σ | [2] |
| 1090 | ν(C–O–C)_glycosidic, asymm_ | Not Identified | σ | [13] |
| 1114 | ν(C–OH) | Monomeric Sugars | §, Δ, σ, ø | [20] |
| 1118 | ν(C–O–C)_glycosidic, symm_ | Not Identified | §, Δ, σ, ø | [13] |
|  | ν(C_b_CH_3_), ν(C_a_C_b_N)_IV_ | Chlorophyll-a |  | [6] |
| 1128 | δ(C–O–C) + δ(C–C) | Xylan | §, Δ, σ, ø | [10] |
| 1147 | ν(C_a_N) | Pheophytin-a | §, Δ, σ, ø | [21] |
|  | ν(C_a_N)_II_, ν(C_b_CH_3_), δ(C_a_NC_a_)_I_ | Chlorophyll-a |  | [6] |
| 1157 | ν(C–C) | Carotenoids | §, Δ, σ, ø | [5, 15] |
|  | ν(C_a_N) | Chlorophyll-a |  | [21] |
|  |  | Pheophytin-a |  |  |
| 1187 | ν(C–N) | Chlorophyll | §, Δ, σ, ø | [1] |
|  | ν(C–O–H)_aromatic_ | Lignin |  | [22] |
| 1218 | Not Identified | Lignin | §, Δ, σ, ø | [10] |
|  | Not Identified | Xylan |  |  |
| 1226 | δ(C_m_H) | Chlorophyll-a | §, Δ, σ, ø | [21] |
| 1254 | δ(C–H) | Pectins | ø | [2] |
| 1264 | Not Identified | 2-deoxy-D-erythropentose | Δ | [7] |
| 1267 | Not Identified | Lignin | §, Δ, σ | [22] |
| 1272 | CH_2_–O–H related | α-D-glucose | §, Δ, σ, ø | [23, 24] |
| 1279 | δ(CH_3_), τ(CH_3_) | Not Identified | σ, ø | [13] |
| 1288 | δ(CH_2_, CH_3_) | Aliphatics | §, Δ, σ, ø | [5, 25] |
| 1303 | Not Identified | 2-deoxy-D-erythropentose | Δ, σ, ø | [7] |
| 1308 | Not Identified | Carotenoids | §, Δ, σ, ø | [26] |
| 1310 | ν(CH_2_) | Not Identified | §, Δ, σ, ø | [13] |
| 1322 | δ(CH_3_) | Not Identified | σ, ø | [13] |
| 1328 | δ(CH_3_) | Pectins | §, Δ, σ, ø | [2, 5] |
|  | ν(C_a_N) | Chlorophyll-a |  | [21] |
| 1343 | δ(CH_2_, CH_3_) | α-amyrin | §, Δ, σ, ø | [25] |
|  | ν(C_a_N) | Chlorophyll-a |  | [21] |
| 1355 | δ(CH_2_, CH_3_) | Aliphatics | §, Δ, σ, ø | [5, 25] |
|  | ν(C_a_C_b_) | Chlorophyll-a |  | [21] |
| 1361 | ν(C_a_C_b_) | Pheophytin-a | ø | [21] |
| 1370 | Not Identified | Glucomannan | §, Δ, σ, ø | [10] |
| 1378 | ν(C_a_C_b_) | Chlorophyll-a | § | [21] |
|  |  | Pheophytin-a |  |  |
|  | δ(CH), δ(OH)_in-plane_ | Xylan |  | [12] |
| 1383 | Not Identified | 2-deoxy-D-erythropentose | §, Δ, σ | [7] |
| 1386 | ν(C_a_C_m_) | Pheophytin-a | Δ, ø | [21] |
| 1389 | Not Identified | Carotenoids | §, σ, ø | [27] |
| 1394 | Not Identified | 2-deoxy-D-erythropentose | §, Δ, σ | [7] |
|  | ν(C_a_C_b_)_II_, ν(C_a_C_m_)_α, β_ | Chlorophyll-a |  | [6] |
| 1416 | δ(CH_2_) | Not Identified | ø | [13] |
| 1439 | δ(CH_3_)_asymm_ | Aliphatics | §, Δ, σ, ø | [25] |
| 1456 | δ(CH_2_, CH_3_) | α-amyrin | §, Δ, σ, ø | [25] |
| 1462 | ν_s_(CH_2_) | Cellulose (amorphous) | §, Δ, σ, ø | [11, 28] |
| 1467 | δ(CH_2_, CH_3_) | Aliphatics | §, σ | [25] |
| 1473 | Unknown | 2-deoxy-D-erythropentose | Δ, σ, ø | [7] |
| 1481 | ν_s_(CH_2_) | Cellulose (crystalline) | §, Δ, σ, ø | [11, 28] |
| 1487 | δ(CH_2_, CH_3_) | Aliphatics | §, Δ, σ, ø | [25] |
|  | ν(C_a_C_m_) | Chlorophyll-a |  | [21] |
| 1494 | ν(C_a_C_m_) | Pheophytin-a | §, Δ, σ, ø | [21] |
| 1525 | ν(C=C) | Carotenoids | §, Δ, σ, ø | [5, 15] |
| 1553 | ν(C_a_C_b_) | Chlorophyll-a | §, Δ, σ, ø | [1] |
| 1581 | ν(C_a_C_m_) | Chlorophyll-a | ø | [21] |
|  |  | Pheophytin-a |  |  |
| 1606 | ν(C–C)_aromatic_ | Lignin | §, Δ, σ, ø | [5, 29] |
|  | ν(COO–)_asymm_ | Pectins |  | [2] |


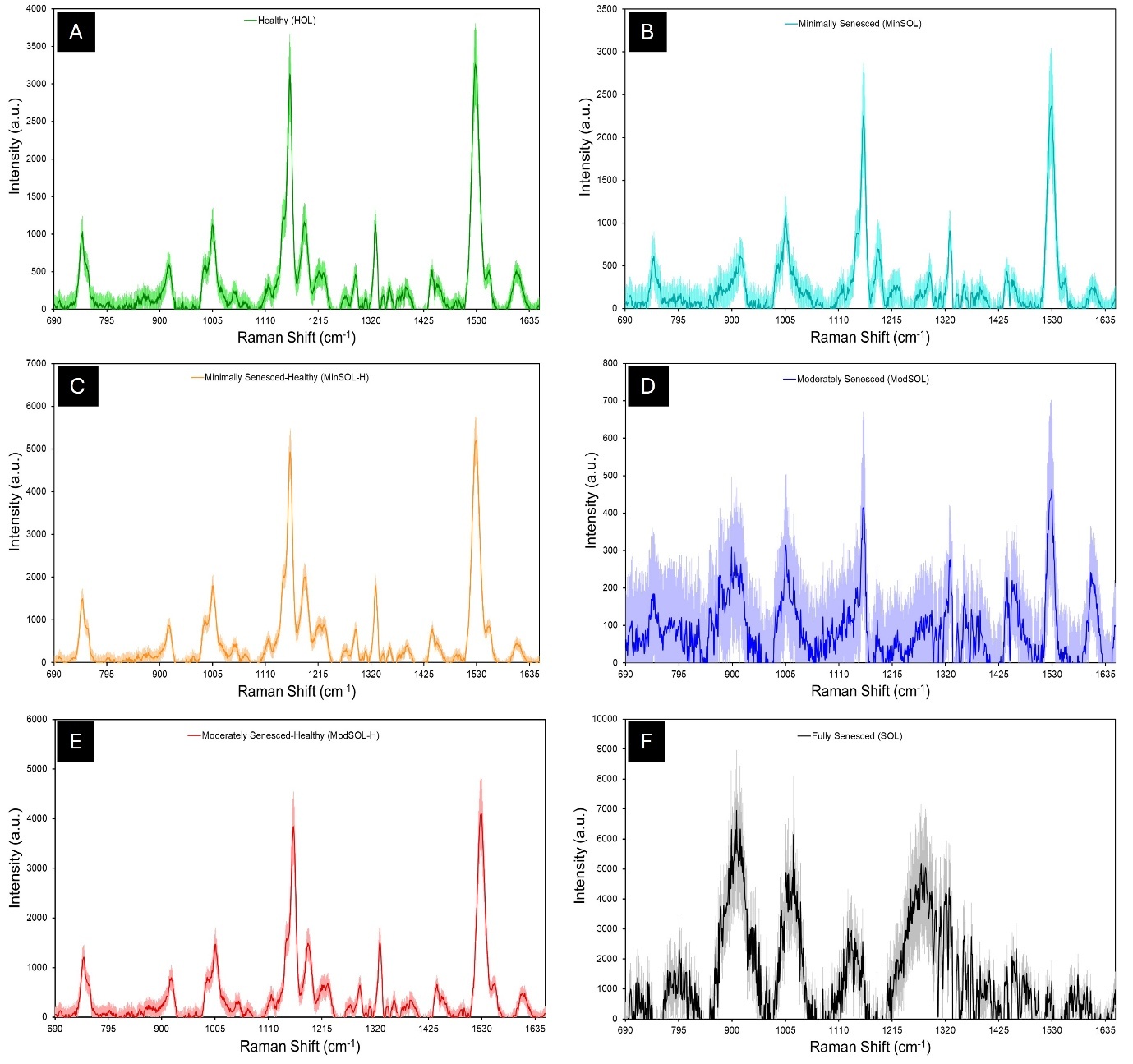


**Figure S1: Individual Leaf Class Senescence Class Spectra.** Molecular profiling using Raman spectroscopic fingerprinting of **(A)** healthy [HOL], **(B)** minimally senescence [MinSOL], **(C)** minimally senescence-healthy [MinSOL-H], **(D)** moderately senesced [ModSOL], **(E)** moderately senesced-healthy [ModSOL-H] and **(F)** fully senesced [SOL] tissue.


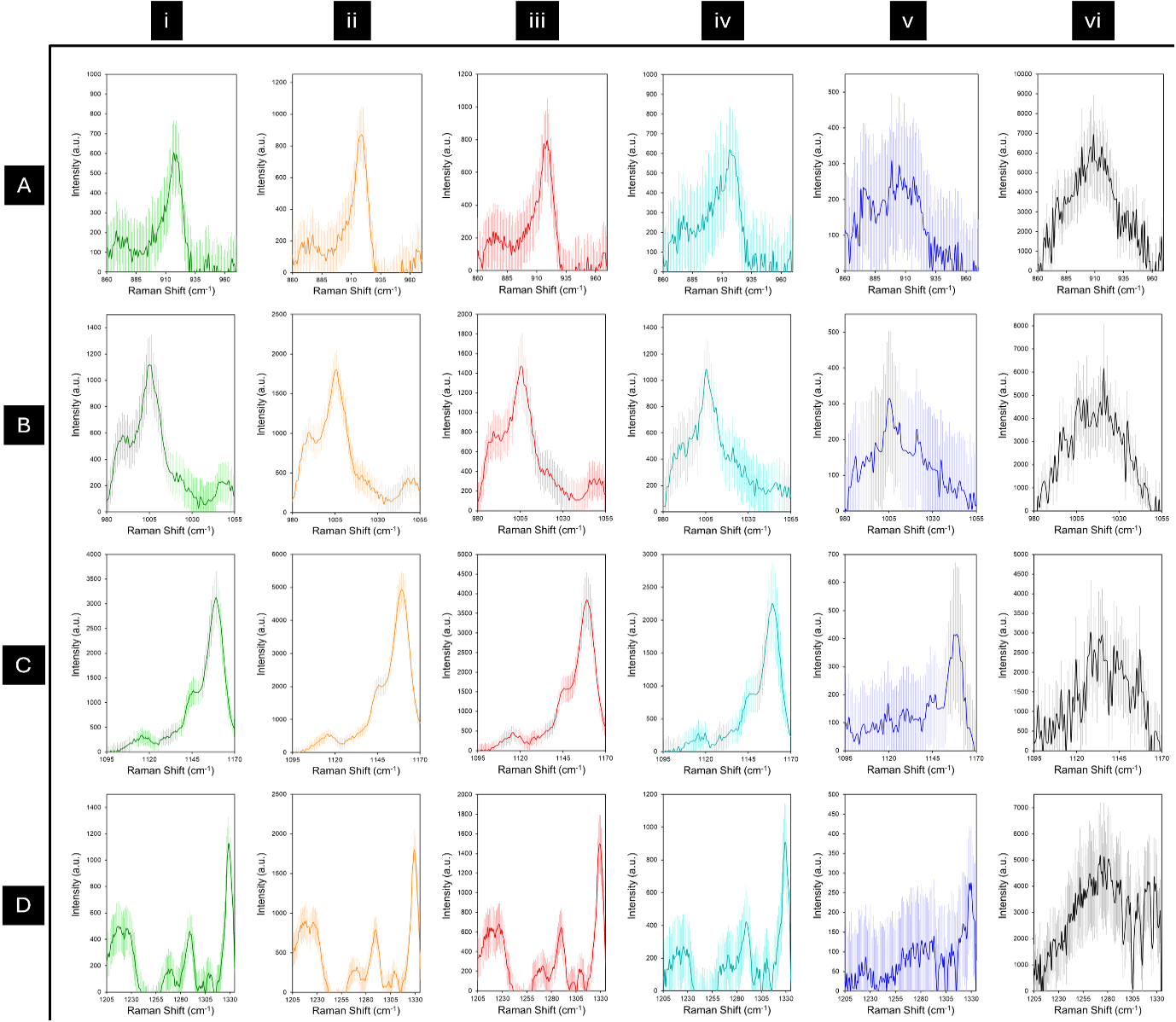


**Figure S2:** **Identified Regions of Interest in Spectral Profiles. (A)** 860-970 cm^-1^. **(B)** 980-1055 cm^-1^. **(C)** 1095-1170 cm^-1^. **(D)** 1205-1335 cm^-1^ for non-senescent [HOL] **(i)**, minimally senesced-healthy [MinSOL-H] **(ii)**, moderately senesced-healthy [ModSOL-H] **(iii)**, minimally senesced [MinSOL] **(iv)**, moderately senesced [ModSOL] **(v)** and fully senesced [SOL] **(vi)** tissue.


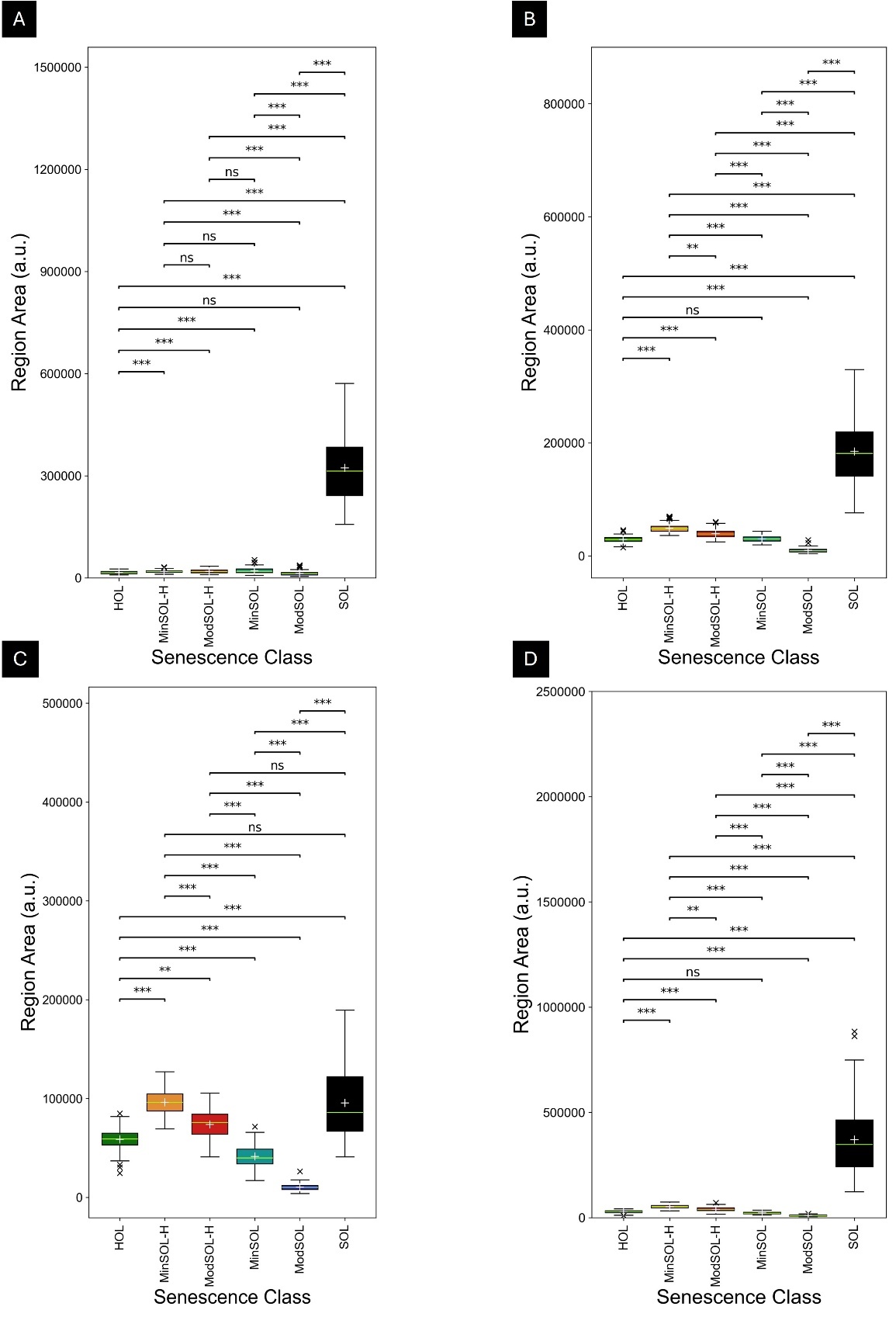


**Figure S3:** **Area-Under-the-Curve for Identified Regions of Interest in Spectral Profiles. (A)** 860-970 cm^-1^. **(B)** 980-1055 cm^-1^. **(C)** 1095-1170 cm^-1^. **(D)** 1205-1335 cm^-1^ for non-senescent [HOL] **(i)**, minimally senesced-healthy [MinSOL-H] **(ii)**, moderately senesced-healthy [ModSOL-H] **(iii)**, minimally senesced [MinSOL] **(iv)**, moderately senesced [ModSOL] **(v)** and fully senesced [SOL] **(vi)** tissue. Brackets represent significantly different pairs of region area-under-the-curves compared via a Kruskal-Wallis ANOVA followed up with a Dunn’s *post-hoc* test with ns>0.05, **p<0.01 and ***p<0.005.


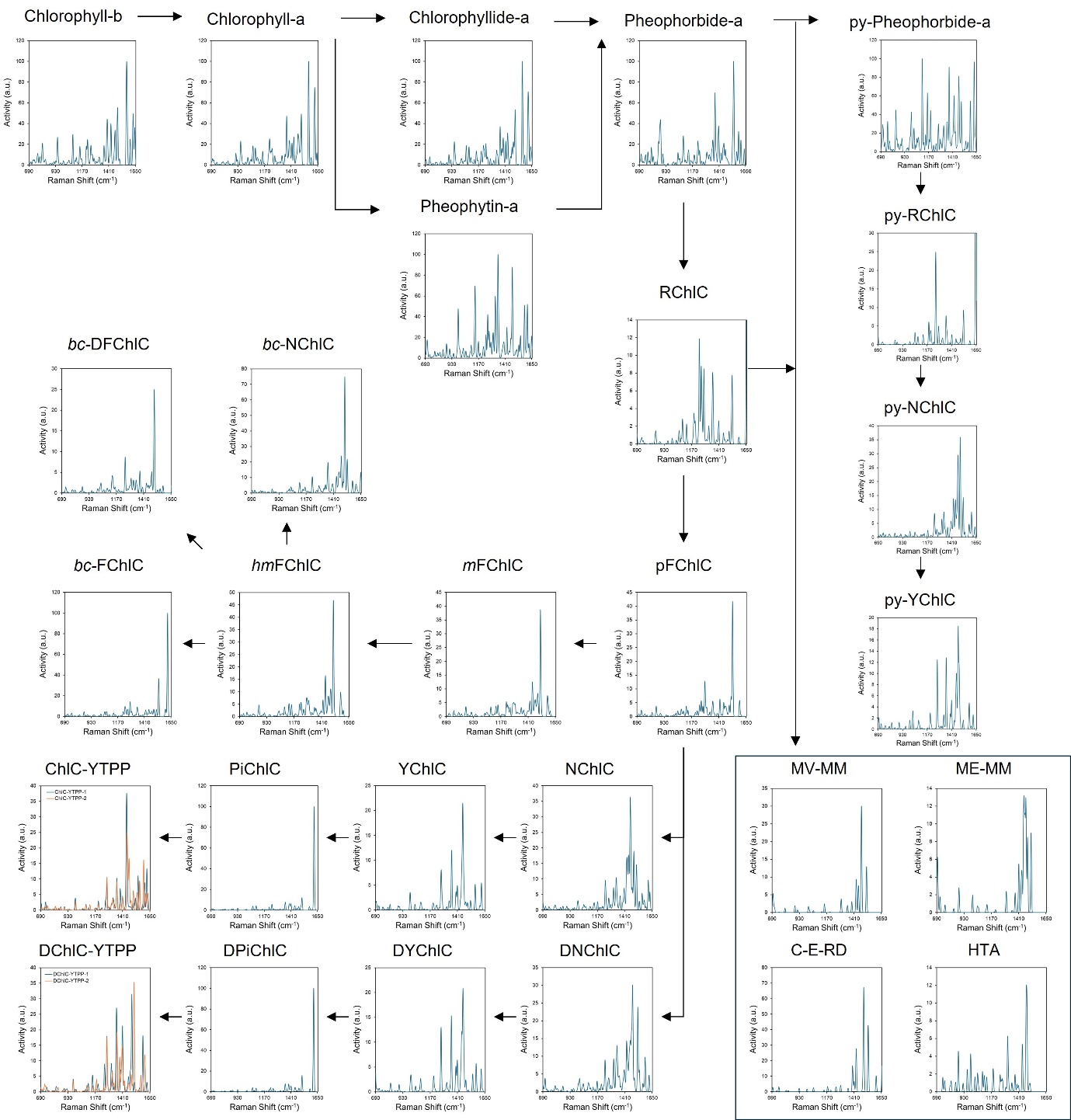


**Figure S4: Simulated Raman Spectra Mechanism of Chlorophyll Catabolism.** MV-MM: methylvinyl maleimide; ME-MM: methylethyl maleimide; C-E-RD: C-E ring derivative; HTA: hematinic acid. Abbreviations starting with D: dioxobilin-type compounds, abbreviations starting with py-: pyro compounds post-decarboxylation of the methyl ester functional group and abbreviations starting with bc-: bicyclic chlorophyll catabolites. For all other abbreviations, see text.


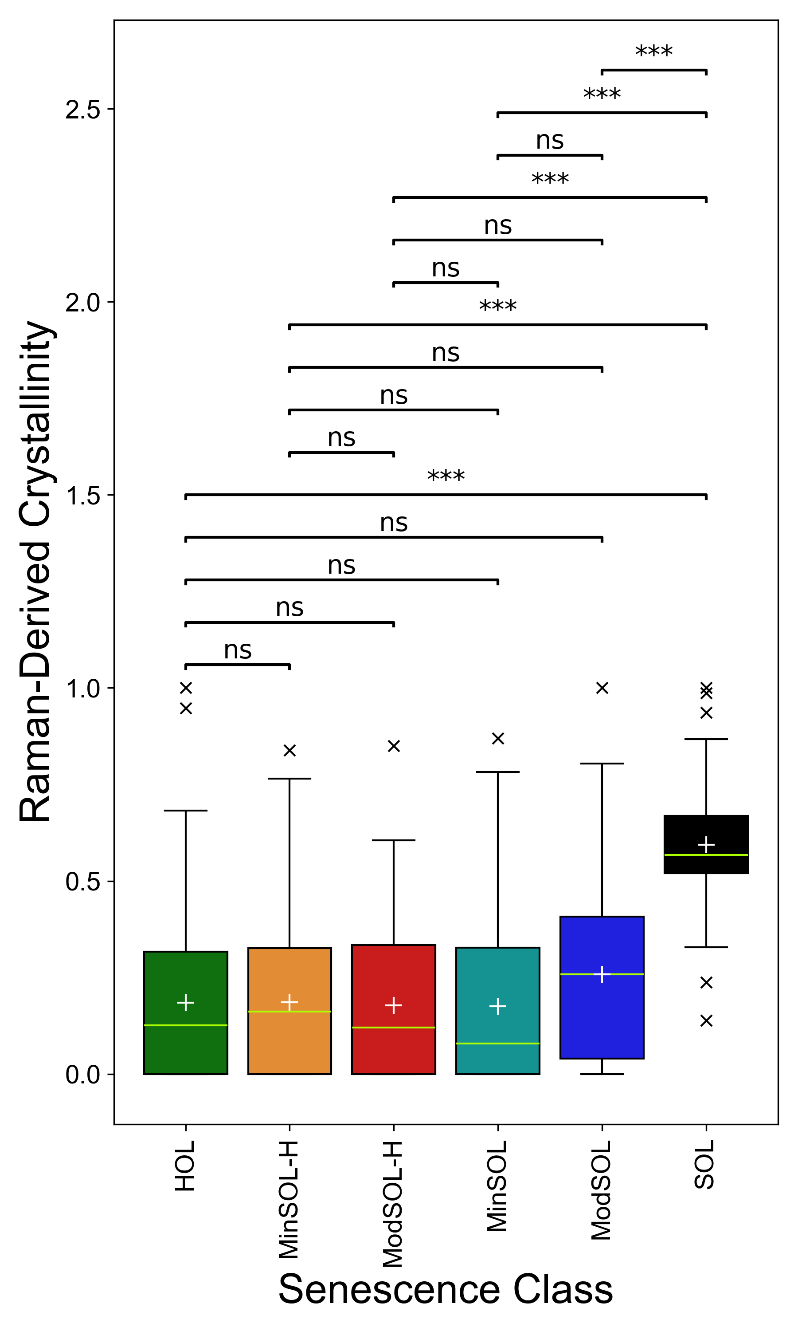


**Figure S5: Raman-Derived Crystallinity Plot.** Crystallinity of non-senescent [HOL], minimally senesced-healthy [MinSOL-H], moderately senesced-healthy [ModSOL-H], minimally senesced [MinSOL], moderately senesced [ModSOL] and fully senesced [SOL] leaf tissue determined using the intensities of the 1462 and 1481 cm^-1^ peaks, as described by Schenzel, Fischer and Brendler [30]. Brackets denote significantly different pairs of Raman-derived crystallinities compared via a Kruskal-Wallis ANOVA followed up with a Dunn’s post-hoc test with ns>0.05 and ***p<0.005.


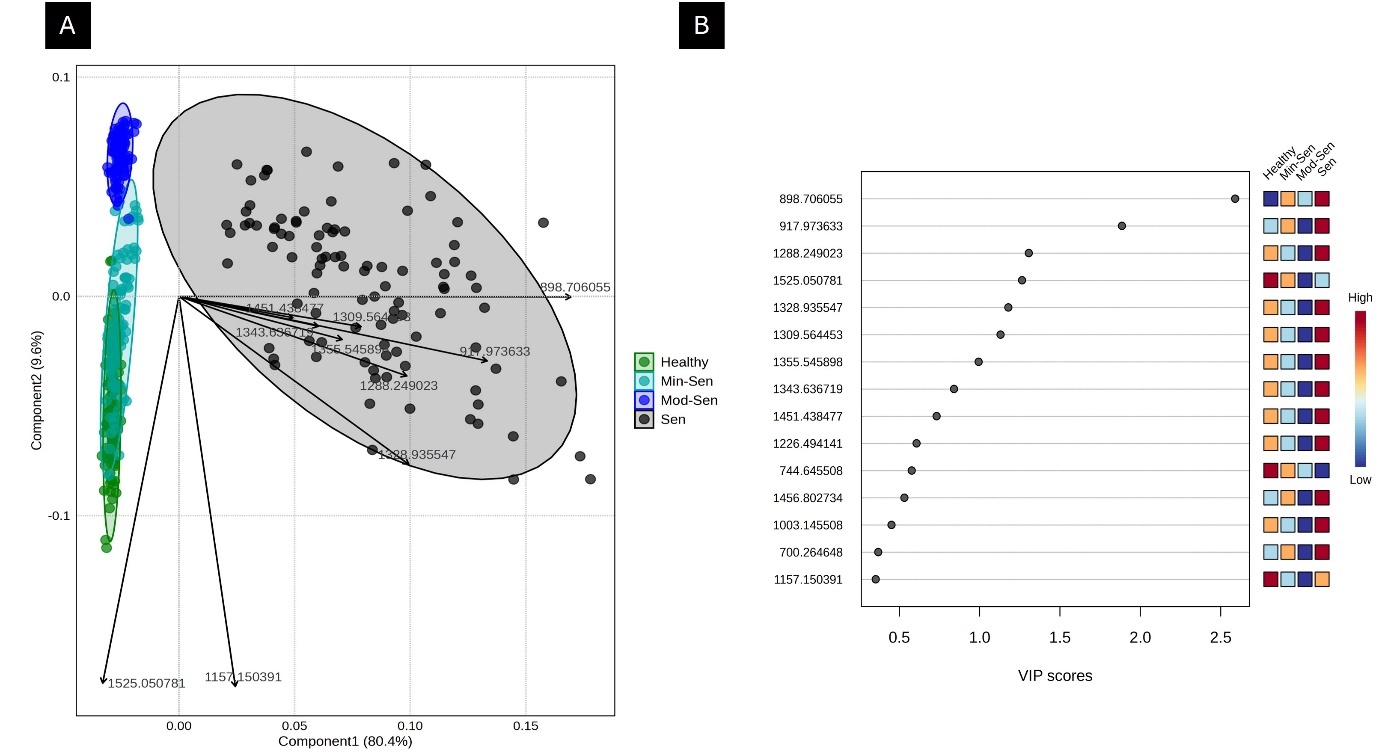


**Figure S6: SOL-Included PLS-DA Biplot and Variable Importance Plots.** The SOL-included PLS-DA biplot **(A)**, performance measured by Q^2^ = 0.87, R^2^ = 0.88 and overall accuracy = 0.74, and variable importance plot **(B)**.


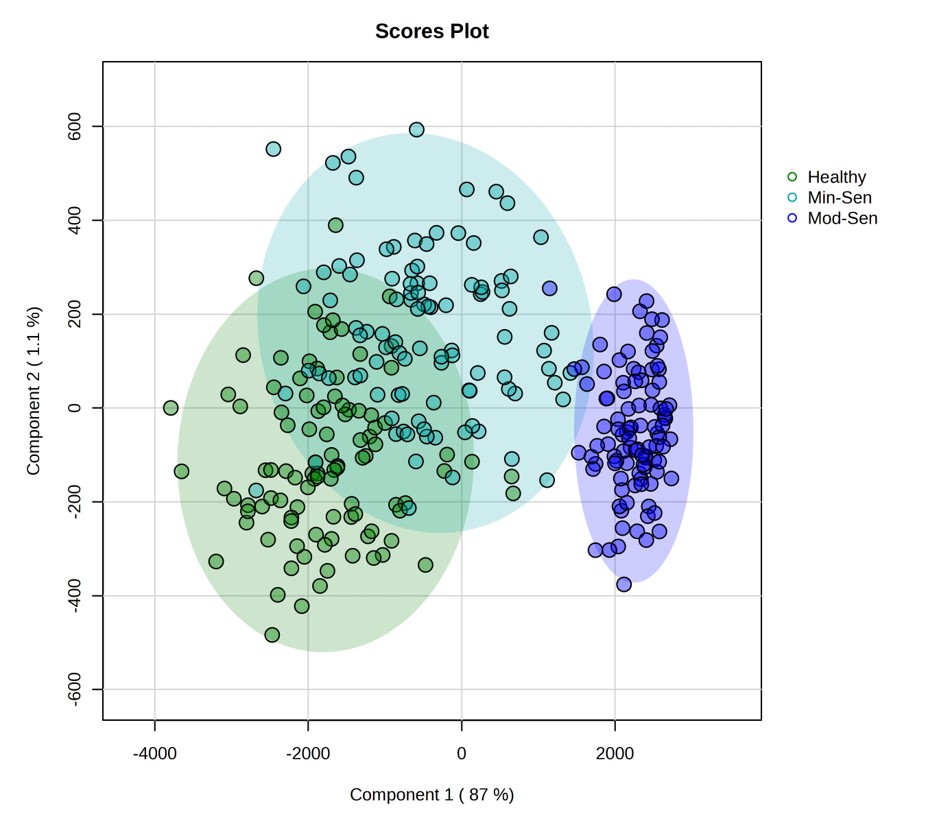


**Figure S7: SOL-Excluded PLS-DA.** SOL-excluded PLS-DA based on 5 component search and 5-fold cross validation method with performance measured by Q^2^ = 0.81, R^2^ = 0.83 and overall accuracy = 0.87**.**


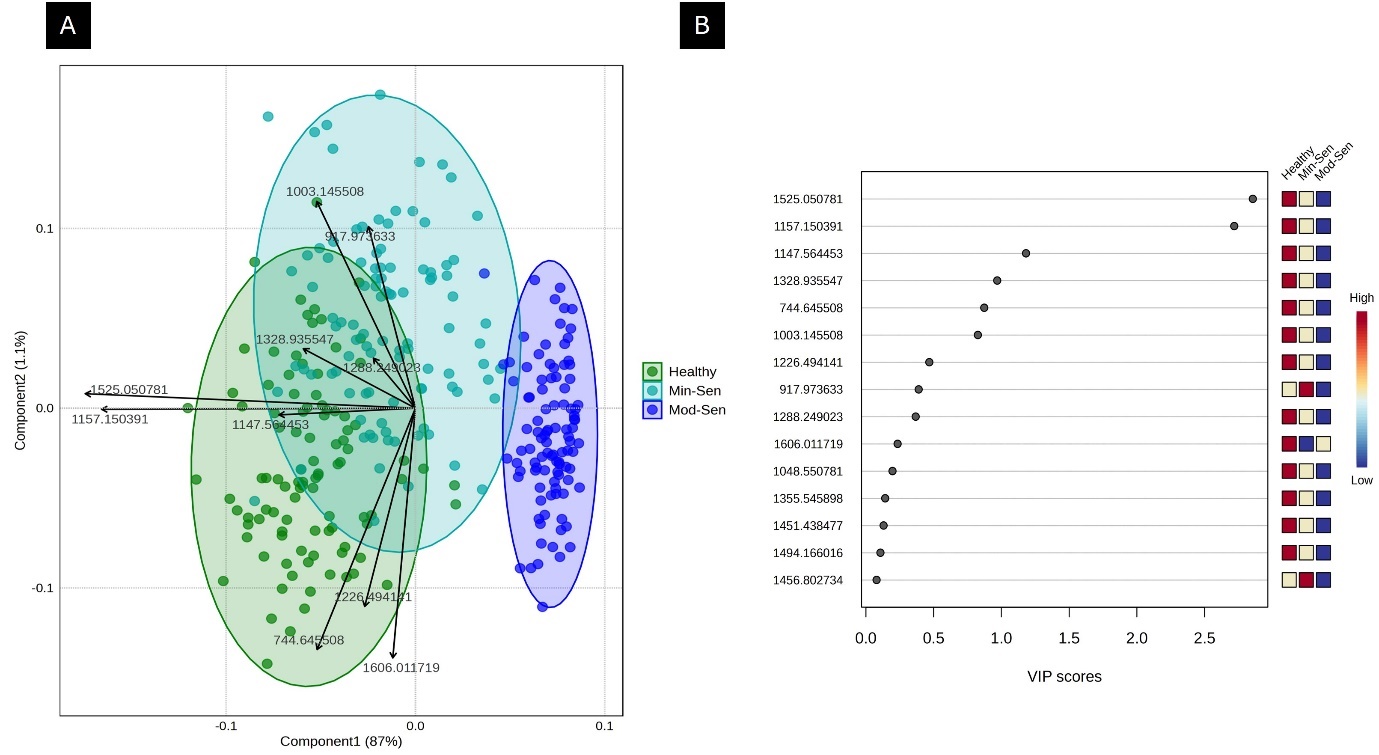


**Figure S8: SOL-Excluded PLS-DA Biplot and Variable Importance Plots.** The SOL-excluded PLS-DA biplot, with performance measured by Q^2^ = 0.81, R^2^ = 0.83 and overall accuracy = 0.87 **(A)** and variable importance plot **(B)**.


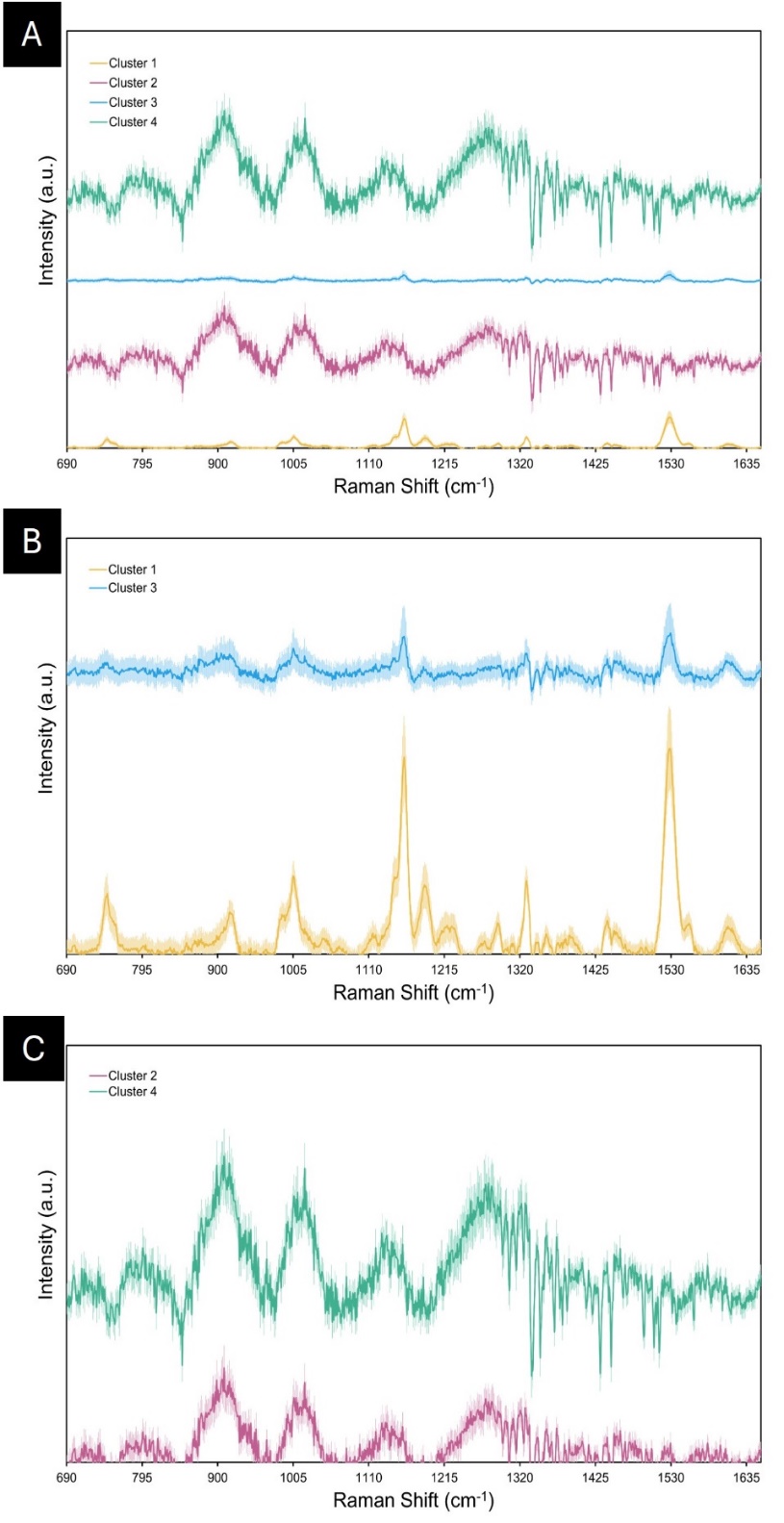


**Figure S9: K-means Cluster Analysis.** The stacked plot for the overall averages of Clusters 1-4 **(A)** with individual stacked plots for Clusters 1 and 3 **(B)** and Clusters 2 and 4 **(C)**


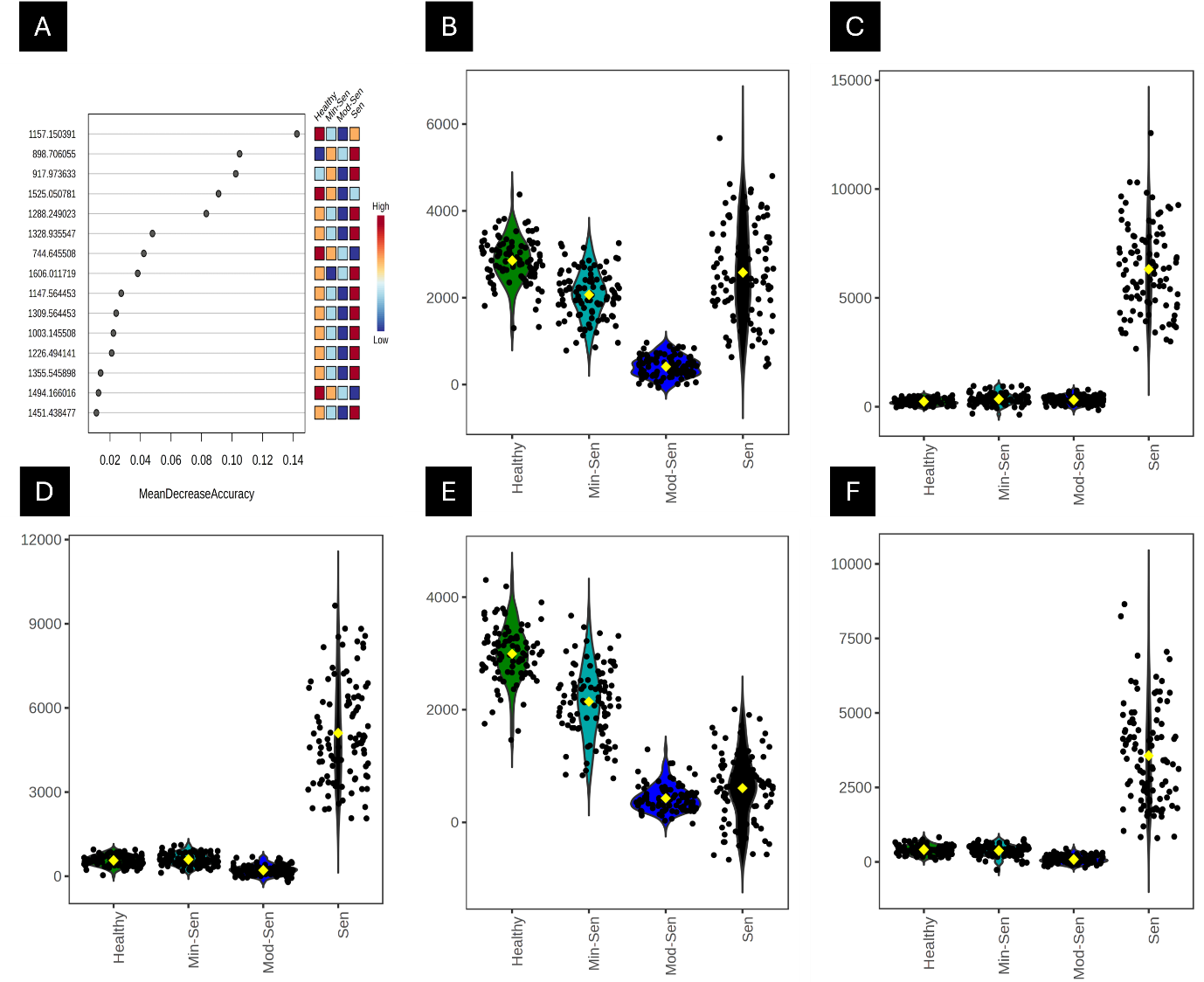


**Figure S10:** **Random Forest Analysis.** The variable importance plot for the random forest model **(A)** with individual violin plots showing the distribution of peak intensity (a.u.) of the top-5 peaks: 1157 **(B)**, 917 **(C)**, 898 **(D)**, 1525 **(E)** and 1288 cm^-1^ **(F)** for healthy (HOL), minimally senesced (Min-Sen, MinSOL), moderately senesced (Mod-Sen, ModSOL) and fully senesced (Sen, SOL) oak leaf tissue**.**


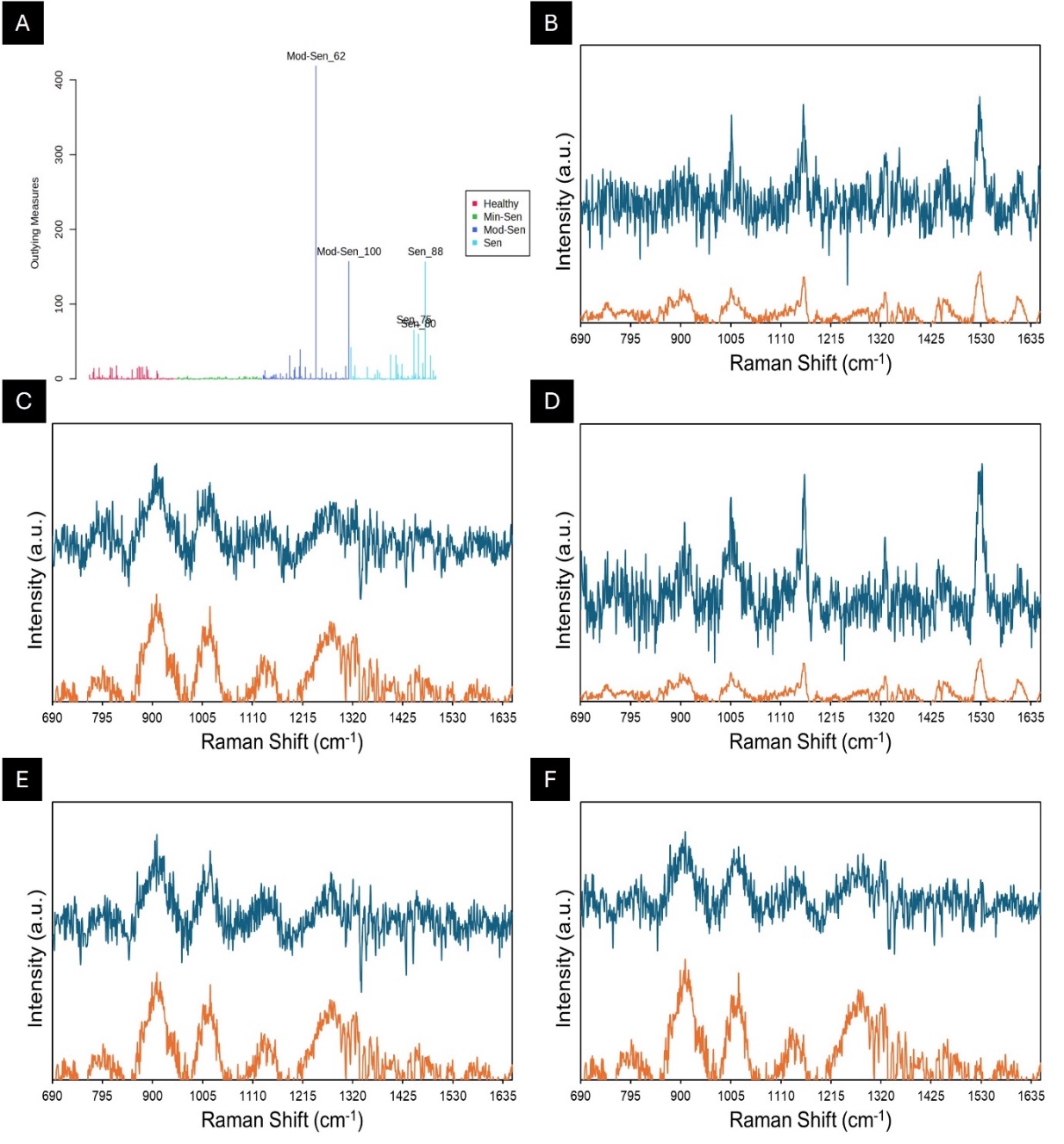


**Figure S11**: **Random Forest Outlier Analysis.** The overall outlier plot **(A)** and Raman spectra comparing sample ModSOL-62 against ModSOL average **(B)**; sample SOL-88 against SOL average **(C)**; sample ModSOL-100 against ModSOL average **(D)**; sample SOL-80 against SOL average **(E)**; and sample SOL-75 against SOL average **(F)**. Samples are shown as blue, averages as orange.


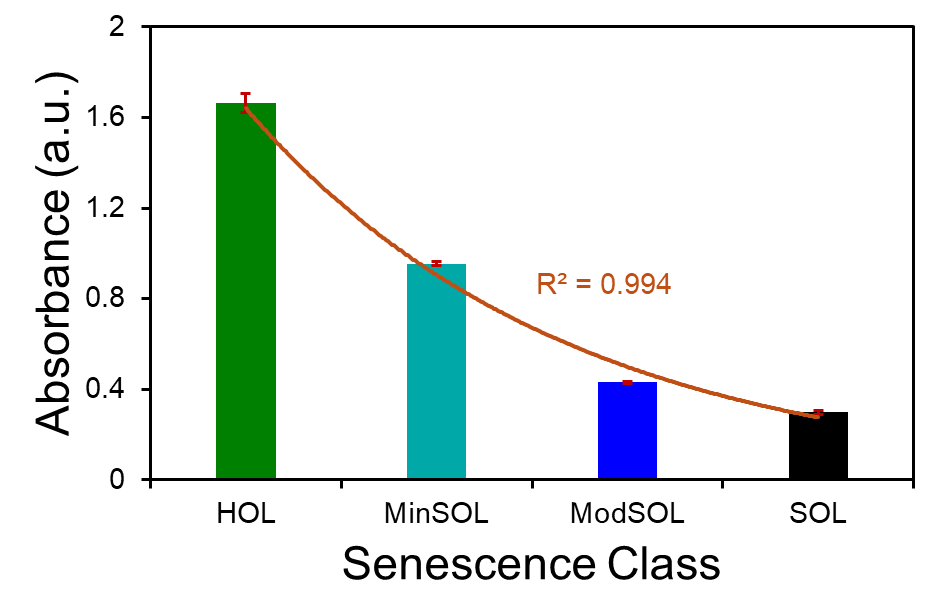


**Figure S12:** **Spectrophotometric Analysis and Trend.** Spectrophotometric absorbance measured at 600 nm for non-senescent [HOL], minimally senesced [MinSOL], moderately senesced [ModSOL] and fully senesced [SOL] leaves. HOL-SOL (p=0.0134) was denoted as the sole significant differentiator between datasets. Remaining classes were devoid of any statistical significance. Bar chart presented with ±SD and with exponential line-of-best-fit (R^2^=0.994).


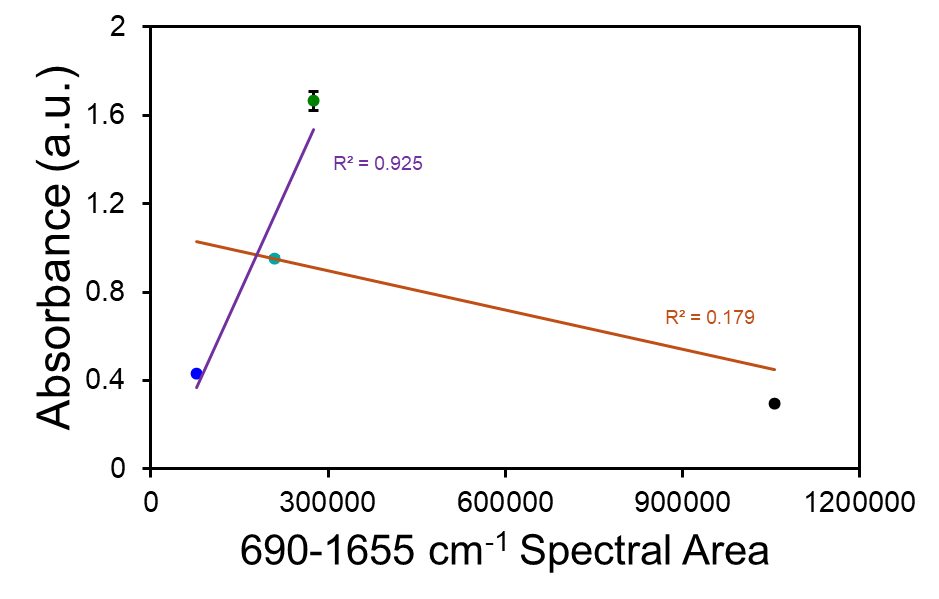


**Figure S13:** **Area-Under-the-Curve-Spectrophotometric Absorbance Trend Analysis.** Correlation of whole spectral range area-under-the-curve against spectrophotometric absorbance measured at 600 nm for non-senescent [Green - HOL], minimally senesced [Teal - MinSOL], moderately senesced [Blue - ModSOL] and fully senesced [Black - SOL] tissue. Scatter chart presented with ±SD and with linear line-of-best-fit, SOL-excluded R^2^=0.925, SOL-included R^2^=0.179.


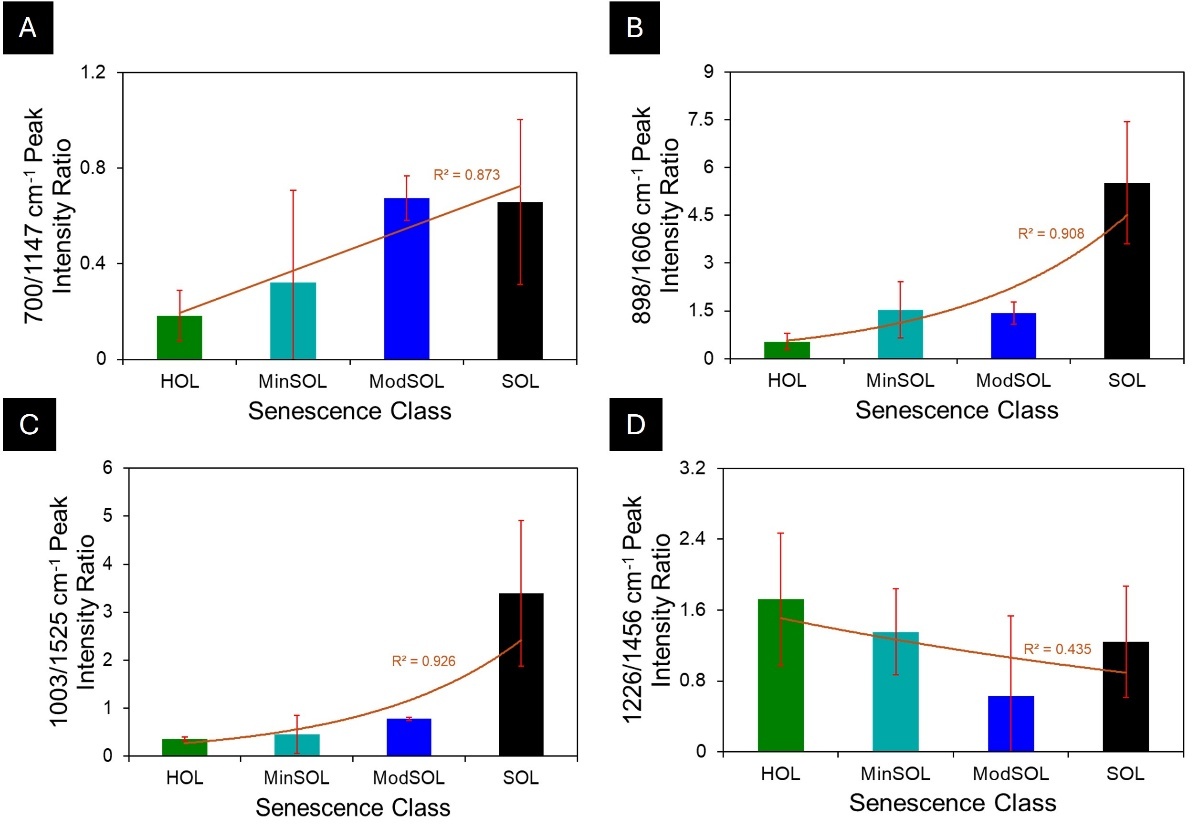


**Figure S14.** **Peak Intensity Ratio Distribution and Trends.** Ratio analysis using bar charts of the (**A**) 700/1147 cm^-1^, (**B**) 898/1606 cm^-1^, (**C**) 1003/1525 cm^-1^ and (**D**) 1226/1456 cm^-1^ Raman peak intensity ratios each with lines of best fit showing R^2^ values of: 0.873, 0.908, 0.926 and 0.435 respectively.

*
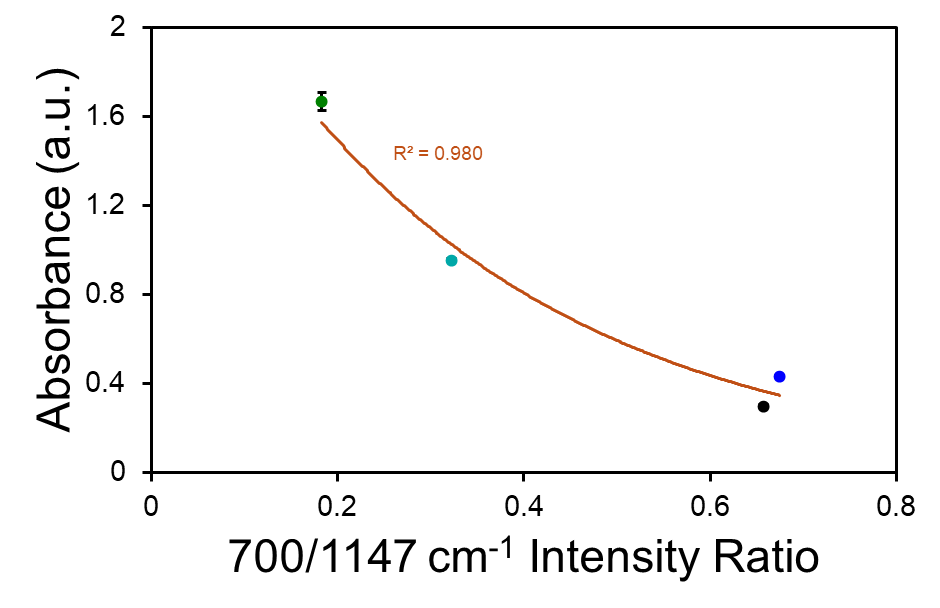
*

**Figure S15: 700/1147 cm-^1^ Peak Intensity Ratio-Spectrophotometric Absorbance Trend Analysis.** Correlation of 700/1147 cm^-1^ peak intensity ratio against spectrophotometric absorbance measured at 600 nm for non-senescent [Green - HOL], minimally senesced [Teal - MinSOL], moderately senesced [Blue - ModSOL] and fully senesced [Black - SOL] tissue. Scatter chart presented with ±SD and with exponential line-of-best-fit, R^2^=0.980.

**References**

1. Koyama Y, Umemoto Y, Akamatsu A, Uehara K, Tanaka M: **Raman spectra of chlorophyll forms**. *Journal of Molecular Structure* 1986, **146**:273-287.

2. Synytsya A, Čopı́ková J, Matějka P, Machovič V: **Fourier transform Raman and infrared spectroscopy of pectins**. *Carbohydrate Polymers* 2003, **54**(1):97-106.

3. Jorge Villar SE, Edwards HGM: **Raman spectroscopy in astrobiology**. *Analytical and Bioanalytical Chemistry* 2006, **384**(1):100-113.

4. Trebolazabala J, Maguregui M, Morillas H, de Diego A, Madariaga JM: **Portable Raman spectroscopy for an in-situ monitoring the ripening of tomato (Solanum lycopersicum) fruits**. *Spectrochimica Acta Part A: Molecular and Biomolecular Spectroscopy* 2017, **180**:138-143.

5. Farber C, Shires M, Ong K, Byrne D, Kurouski D: **Raman spectroscopy as an early detection tool for rose rosette infection**. *Planta* 2019, **250**(4):1247-1254.

6. Boldt NJ, Donohoe RJ, Birge RR, Bocian DF: **Chlorophyll model compounds: effects of low symmetry on the resonance Raman spectra and normal mode descriptions of nickel(II) dihydroporphyrins**. *Journal of the American Chemical Society* 1987, **109**(8):2284-2298.

7. Mathlouthi M, Seuvre AM, Koenig JL: **F.T.-I.R. and laser-raman spectra of d-ribose and 2-deoxy-d-erythro-pentose (“2-deoxy-d-ribose”)**. *Carbohydrate Research* 1983, **122**(1):31-47.

8. Clark KR, Goldberg Oppenheimer P: **Vibrational spectroscopic profiling of biomolecular interactions between oak powdery mildew and oak leaves**. *Soft Matter* 2024.

9. Schulz H, Baranska M: **Identification and quantification of valuable plant substances by IR and Raman spectroscopy**. *Vibrational Spectroscopy* 2007, **43**(1):13-25.

10. Agarwal UP, Ralph SA: **FT-Raman Spectroscopy of Wood: Identifying Contributions of Lignin and Carbohydrate Polymers in the Spectrum of Black Spruce (Picea Mariana)**. *Applied Spectroscopy* 1997, **51**(11):1648-1655.

11. Szymańska-Chargot M, Cybulska J, Zdunek A: **Sensing the Structural Differences in Cellulose from Apple and Bacterial Cell Wall Materials by Raman and FT-IR Spectroscopy**. In: *Sensors.* vol. 11; 2011: 5543-5560.

12. Kačuráková M, Wellner N, Ebringerová A, Hromádková Z, Wilson RH, Belton PS: **Characterisation of xylan-type polysaccharides and associated cell wall components by FT-IR and FT-Raman spectroscopies**. *Food Hydrocolloids* 1999, **13**(1):35-41.

13. Edwards HGM, Farwell DW, Webster D: **FT Raman microscopy of untreated natural plant fibres**. *Spectrochimica Acta Part A: Molecular and Biomolecular Spectroscopy* 1997, **53**(13):2383-2392.

14. Prinsloo LC, du Plooy W, van der Merwe C: **Raman spectroscopic study of the epicuticular wax layer of mature mango (Mangifera indica) fruit**. *Journal of Raman Spectroscopy* 2004, **35**(7):561-567.

15. Gill D, Kilponen RG, Rimai L: **Resonance Raman Scattering of Laser Radiation by Vibrational Modes of Carotenoid Pigment Molecules in Intact Plant Tissues**. *Nature* 1970, **227**(5259):743-744.

16. Pérez MRV, Mendoza MGG, Elías MGR, González FJ, Contreras HRN, Servín CC: **Raman Spectroscopy an Option for the Early Detection of Citrus Huanglongbing**. *Applied Spectroscopy* 2016, **70**(5):829-839.

17. Veronelli M, Zerbi G, Stradi R: **In situ resonance Raman spectra of carotenoids in bird's feathers**. *Journal of Raman Spectroscopy* 1995, **26**(8-9):683-692.

18. Tarantilis PA, Beljebbar A, Manfait M, Polissiou M: **FT-IR, FT-Raman spectroscopic study of carotenoids from saffron (Crocus sativus L.) and some derivatives**. *Spectrochimica Acta Part A: Molecular and Biomolecular Spectroscopy* 1998, **54**(4):651-657.

19. Wiley JH, Atalla RH: **Band assignments in the raman spectra of celluloses**. *Carbohydrate Research* 1987, **160**:113-129.

20. Mandrile L, Rotunno S, Miozzi L, Vaira AM, Giovannozzi AM, Rossi AM, Noris E: **Nondestructive Raman Spectroscopy as a Tool for Early Detection and Discrimination of the Infection of Tomato Plants by Two Economically Important Viruses**. *Analytical Chemistry* 2019, **91**(14):9025-9031.

21. Perng JH, Bocian DF: **Resonance Raman spectra of chlorin and chlorophyll radical anions**. *The Journal of Physical Chemistry* 1992, **96**(25):10234-10240.

22. Agarwal UP: **1064 nm FT-Raman spectroscopy for investigations of plant cell walls and other biomass materials**. *Front Plant Sci* 2014, **5**:490.

23. Vasko PD, Blackwell J, Koenig JL: **Infrared and raman spectroscopy of carbohydrates.: Part II: Normal coordinate analysis of α-D-glucose**. *Carbohydrate Research* 1972, **23**(3):407-416.

24. Cael SJ, Koenig JL, Blackwell J: **Infrared and raman spectroscopy of carbohydrates: Part III: raman spectra of the polymorphic forms of amylose**. *Carbohydrate Research* 1973, **29**(1):123-134.

25. Yu MML, Schulze HG, Jetter R, Blades MW, Turner RFB: **Raman Microspectroscopic Analysis of Triterpenoids Found in Plant Cuticles**. *Applied Spectroscopy* 2007, **61**(1):32-37.

26. Rimai L, Heyde ME, Gill D: **Vibrational spectra of some carotenoids and related linear polyenes. Raman spectroscopic study**. *Journal of the American Chemical Society* 1973, **95**(14):4493-4501.

27. Pudney PDA, Gambelli L, Gidley MJ: **Confocal Raman Microspectroscopic Study of the Molecular Status of Carotenoids in Tomato Fruits and Foods**. *Applied Spectroscopy* 2011, **65**(2):127-134.

28. Kavkler K, Demšar A: **Examination of cellulose textile fibres in historical objects by micro-Raman spectroscopy**. *Spectrochimica Acta Part A: Molecular and Biomolecular Spectroscopy* 2011, **78**(2):740-746.

29. Agarwal UP: **Raman imaging to investigate ultrastructure and composition of plant cell walls: distribution of lignin and cellulose in black spruce wood (Picea mariana)**. *Planta* 2006, **224**(5):1141-1153.

30. Schenzel K, Fischer S, Brendler E: **New Method for Determining the Degree of Cellulose I Crystallinity by Means of FT Raman Spectroscopy**. *Cellulose* 2005, **12**(3):223-231.
